# Decreased expression of the translation factor eIF3e induces senescence in breast cancer cells via suppression of PARP1 and activation of mTORC1

**DOI:** 10.18632/oncotarget.27923

**Published:** 2021-03-30

**Authors:** Christelle Morris, Sébastien Durand, Pierre Jalinot

**Affiliations:** ^1^University Lyon, ENS de Lyon, Université Claude Bernard Lyon 1, CNRS UMR 5239, INSERM U1210, LBMC, Lyon, France; ^2^University Lyon, Cancer Research Center of Lyon, Université Claude Bernard Lyon 1, INSERM 1052, CNRS UMR 5286, Centre Léon Bérard, Cancer Cell Plasticity Department, Equipe 'Transcriptome Diversity in Stem Cells', Lyon, France

**Keywords:** breast cancer, senescence-associated secretory phenotype, eIF3, PARP1, mTORC1

## Abstract

Altered expression of the translation factor eIF3e is associated with breast cancer occurrence. We have previously shown that eIF3e deficiency leads to an impaired DNA damage response with a marked decrease in DNA repair by homologous recombination. Here, we explored the possibility to exploit this DNA repair defect in targeted cancer therapy using PARP inhibitors. Surprisingly, eIF3e-deficient breast cancer cells are resistant to these drugs, in contrast to BRCA1-deficient cells. Studying this, we found that eIF3e-depleted cells synthesize lowered amounts of PARP1 protein, due to a weakened translation of the corresponding mRNA, associated with a strong decrease in cellular poly(ADP-ribosyl)ation. Additionally, we discovered that the mTORC1 signaling pathway is aberrantly activated in response to eIF3e suppression. Together, these PARP1 and mTORC1 dysfunctions upon eIF3e depletion are causally linked to induction of cellular senescence associated with a pro-inflammatory secretory phenotype. This study provides mechanistic insights into how eIF3e protects against breast cancer, with potential novel cancer therapeutic opportunities. While PARP inhibitors appear as inappropriate drugs for eIF3e-deficient breast tumors, our findings suggest that such cancers may benefit from senolytic drugs or mTORC1 inhibitors.

## INTRODUCTION

Cellular senescence is a major driver of various age-related diseases, including cancer. This complex cell state operates as a barrier against cancer by arresting proliferation of cancerous cells, but it also favors a pro-tumorigenic environment by inducing the secretion of numerous factors collectively referred to as the senescence-associated secretory phenotype (SASP) [1–3]. Additionally, a fraction of cancer cells rendered senescent by DNA-damaging therapy resumes proliferation and represents a tumor-reinitiating cell population in particularly aggressive tumors [4, 5].

One trigger of cellular senescence is DNA damage persistence. The poly(ADP-ribose) polymerase 1 (PARP1) has long been known to regulate the DNA damage response [6, 7] and a decrease of PARP1 enzymatic activity has been linked to senescence [8]. PARP1 is involved in repair of single-strand breaks (SSBs) or double-strand breaks (DSBs), and repair of replication fork damage. Upon DNA binding, PARP1 rapidly synthesizes and attaches poly(ADP-ribose) (PAR) polymers onto itself and various target proteins. These PAR modifications enable the recruitment of repair proteins. PARP1 is also required for genome stability during DNA replication [9, 10]. By exploiting synthetic lethality, PARP inhibitors (PARPi) are used in clinic to treat various cancers defective in DNA repair via homologous recombination (HR), particularly those due to BRCA1/2 loss of functions [11–13].

A second condition that induces senescence is the hyperactivity of an expansion signal fostering either cell growth or proliferation. In particular, activation of the mechanistic target of rapamycin complex 1 (mTORC1) contributes to senescence and SASP response [14, 15]. Activated mTORC1 integrates nutrient status to regulate cell growth and protein synthesis [16, 17]. mTORC1 phosphorylates the eukaryotic translation initiation factor 4E (eIF4E)-binding proteins (4E-BPs) and the ribosomal protein S6 kinase 1 (S6K1). When phosphorylated, S6K1 is released from the eukaryotic translation initiation factor 3 (eIF3) and phosphorylates its downstream targets [18].

eIF3 assembles in humans 13 subunits named eIF3a to eIF3m [19–21]. Several clinical and experimental observations suggest that altered expression of the eIF3e subunit is associated with the occurrence and development of breast cancer [22–27]. However, the role of eIF3e in breast cancer remains to be better understood. In previous studies, we identified that eIF3e deficiency leads to an impaired DNA damage response with a marked decrease in DNA repair via the HR pathway [28, 29]. Consistently, we observed that several DNA repair proteins including ATM and BRCA1/2 fail to accumulate at DSB sites, although eIF3e silencing did not cause obvious changes in the total amounts of these proteins [28, 29]. Based on the connection between reduced eIF3e levels and impaired HR, we hypothesized that eIF3e-deficient breast tumors might be vulnerable to PARPi therapy.

In this study, we explored the possibility that eIF3e might be a synthetic lethal partner of PARP1. We discovered that eIF3e-deficient breast cancer cells are resistant to PARPi. In search of the underlying mechanism, we found that eIF3e deficiency causes reduced PARP1 expression and mTORC1 hyperactivation that drive breast cancer cells into senescence and secretion of inflammatory factors, providing potential novel cancer therapeutic opportunities.

## RESULTS

### eIF3e deficiency renders breast cancer cells refractory to PARP inhibitors

**Figure 1:**
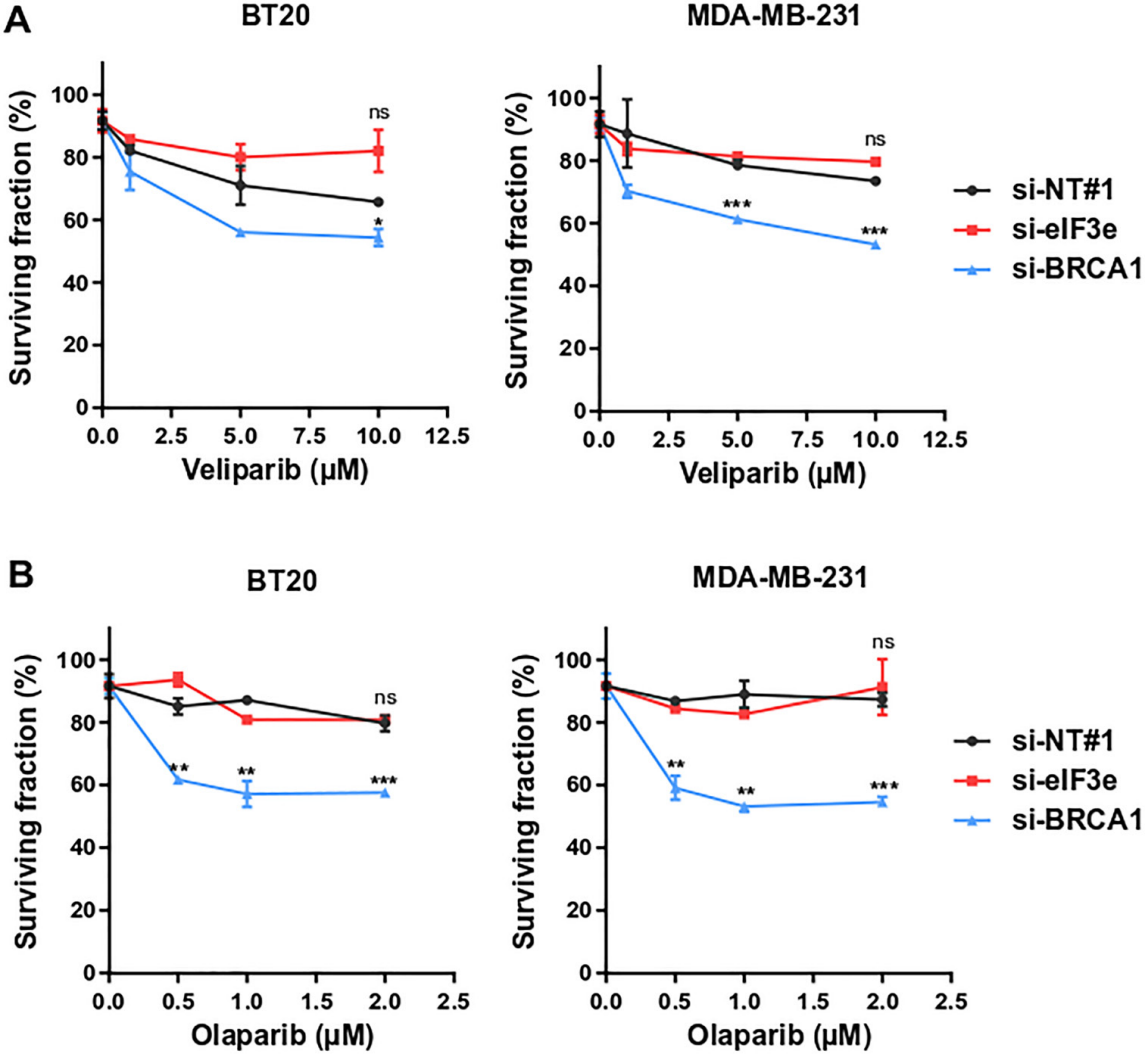
eIF3e knockdown renders breast cancer cells resistant to PARPi. (**A**, **B**) Cell viability assay in response to PARPi veliparib (A) and olaparib (B). BT-20 and MDA-MB-231 cells were transfected with non-targeting siRNA (si-NT#1) or with siRNAs targeting eIF3e (si-eIF3e) or BRCA1 (si-BRCA1). The following day, cells were treated with the indicated concentrations of veliparib or olaparib, and allowed to grow for 4 days before cell proliferation was measured using a cell viability assay. Analysis of cell viability at each drug dose was expressed as a percentage of remaining cells compared to vehicle treated cells. Results from 3 independent experiments. Error bars, means ± SEM. ns, not significant, ^*^ *P* < 0.05, ^**^ *P* < 0.01, ^***^ *P* < 0.001 by unpaired *t* test.

PARPi are showing promise to treat various cancers with HR deficiencies, particularly breast cancers with germline BRCA1/2 mutations [11–13]. Our previous study, primarily performed using HeLa and U2OS cell lines, demonstrated that eIF3e-deficient cancer cells are defective in HR-mediated DNA repair [29]. Therefore, we hypothesized that PARPi therapy could also be effective in treating tumors with low eIF3e expression. Here, we tested the sensitivity of two triple-negative breast cancer cell lines, BT-20 and MDA-MB-231, to the PARPi veliparib, after transfection of either a non-targeting control siRNA or siRNAs targeting eIF3e or BRCA1 as a positive control. Although eIF3e silencing results in HR defect also in breast cancer cells (Supplementary Figure 1), we found that these eIF3e-depleted cells were not sensitive to veliparib treatment, in contrast to BRCA1-depleted cells (Figure 1A). To validate this unexpected result, we tested the sensitivity of eIF3e-depleted cells to olaparib, another clinically approved PARPi. If both olaparib and veliparib display equal catalytic inhibition potency, olaparib, in contrast to veliparib, is much more efficient in trapping PARP1/2 onto DNA [13, 30]. The PARP trapping activity, rather than enzymatic inhibition, is thought to be crucial for cancer cell killing by PARPi. Our assays showed that silencing BRCA1 caused significant cytotoxicity from the lowest olaparib dose, as expected (Figure 1B). However, eIF3e-deficient cells did not respond to olaparib at all tested concentrations, similarly to control cells. These findings indicate that cancer cells with low eIF3e amounts are refractory to PARPi, suggesting that assessing eIF3e levels in tumors could be informative to predict the clinical response to PARPi.

### eIF3e depletion leads to lower levels of PARP1 and PAR polymers

**Figure 2:**
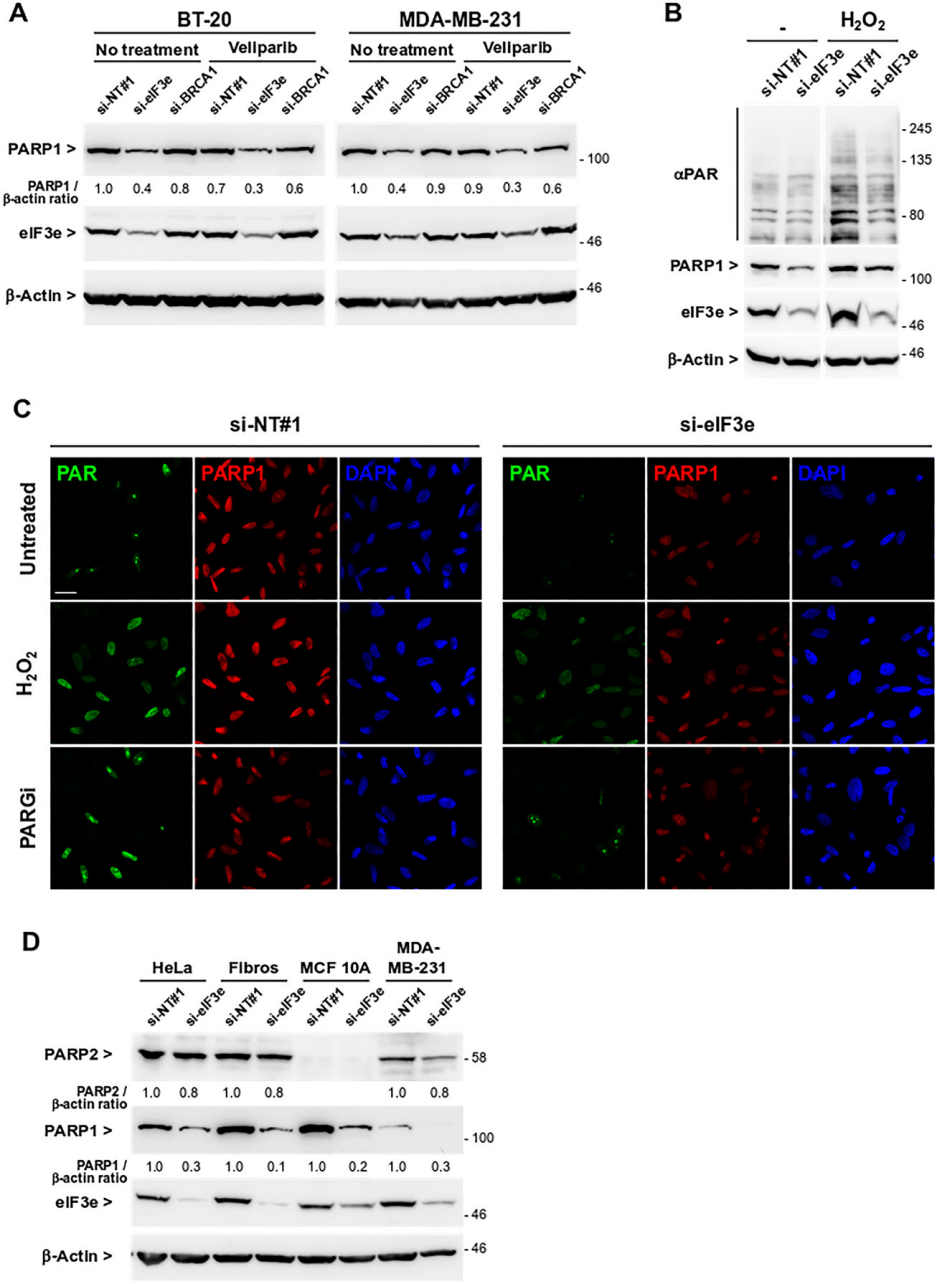
eIF3e depletion leads to lower levels of PARP1 protein and PAR polymers. (**A**) Immunoblots to detect PARP1 protein in total extracts of BT-20 and MDA-MB-231 cells transfected with non-targeting siRNA or with siRNAs targeting eIF3e or BRCA1. One day after siRNA transfection, cells were untreated or treated with 5μM of veliparib for 4 days. Efficiency of eIF3e depletion was determined by detection of eIF3e. Data shown is representative of two independent experiments. (**B**) Immunoblots to detect the level of PAR polymers and PARP1 in total extracts of MDA-MB-231 cells transfected with siRNAs non-targeting or targeting eIF3e. Four days after siRNA transfection, cells were treated or not with 2mM H_2_O_2_ to induce PAR formation, and harvested 5 min later. RNA interference efficacy and equal protein loading were controlled by detection of eIF3e and β-actin, respectively. Data shown correspond to two parts of the same gel and are representative of two independent experiments. (**C**) Immunofluorescence assay to detect PARP1 and PAR chains in MDA-MB-231 cells transfected with control or eIF3e siRNAs. After 4 days, cells were left untreated or treated with 5 μM of the PARG inhibitor PDD 00017273 for 2 h, or with 2 mM H_2_O_2_ for 5 min. Nuclei were counterstained with 4′,6-diamidino-2-phenylindole (DAPI). Shown are representative confocal images of two independent experiments. Scale bar, 20 μM. (**D**) Immunoblots to detect PARP2 and PARP1 in the indicated cell lines transfected with siRNAs non-targeting or targeting eIF3e. Efficiency of eIF3e depletion was determined by detection of eIF3e.

Several mechanisms of resistance to PARPi were described [31], one of them through PARP1 downregulation [30, 32, 33]. PARP1 is needed for proper sensitization to PARPi since these drugs act in part through PARP trapping. To determine whether eIF3e deficiency could modify PARP1 abundance, immunoblots were performed using lysates of BT-20 and MDA-MB-231 cells treated as above. We found that PARP1 levels were markedly reduced after eIF3e knockdown, regardless of the presence or absence of veliparib (Figure 2A). Since PARP1 generates about 80–90% of PAR polymers, we next examined whether this PARP1 downregulation was associated with a decrease in poly(ADP-ribosyl)ation (PARylation). As the basal level of PARylation is very low, cells were subjected where indicated to an oxidative stress with H_2_O_2_ to increase PARP1 basal activity and an immunoblot was performed using an antibody recognizing PAR polymers. We observed a generalized increase in PAR polymers in control siRNA-transfected cells treated with H_2_O_2_, compared to untreated cells (Figure 2B). In contrast, eIF3e-depleted cells exposed to H_2_O_2_ displayed a more limited increase of PAR signals, relative to control cells similarly treated. The impact of eIF3e depletion on PARP1 expression and activity was further studied by immunofluorescence analyses. Where indicated, cells were treated with either H_2_O_2_ to induce PAR formation or an inhibitor of the PAR-degrading enzyme PARG to stabilize PAR polymers. Cells transfected with control siRNA and exposed to each treatment showed a substantial increase in nuclear PAR staining compared to untreated ones (Figure 2C). In contrast, eIF3e-depleted cells did not respond to treatments and displayed reduced levels of both PAR polymers and PARP1 compared to control cells. The fact that PARylation was not reinforced in eIF3e-silenced cells treated with the PARG inhibitor implies that formation rather than degradation of PAR chains is impaired in these cells, which is consistent with a downregulation of PARP1. Since PARP1 and PARP2 have overlapping functions, we assessed whether eIF3e may regulate PARP2 similarly. PARP2 was detected in all cell types tested except MCF 10A, a non-tumorigenic breast cell line, and eIF3e depletion did not grossly alter PARP2 amounts in any of analyzed cells (Figure 2D). In contrast, PARP1 which was expressed in all cell lines was much less abundant upon eIF3e silencing. Together, these results suggest that eIF3e-deficient cells display lower levels of PAR polymers because of reduced expression of PARP1, but not PARP2. These findings provide a molecular basis for the resistance of these cells to PARPi. In addition, by querying a proteomic resource for breast cancer [34], we found a significant positive correlation between the eIF3e and PARP1 protein abundances in the triple-negative breast cancer group (Supplementary Figure 2).

### eIF3e is required for efficient translation of mRNA encoding PARP1

**Figure 3:**
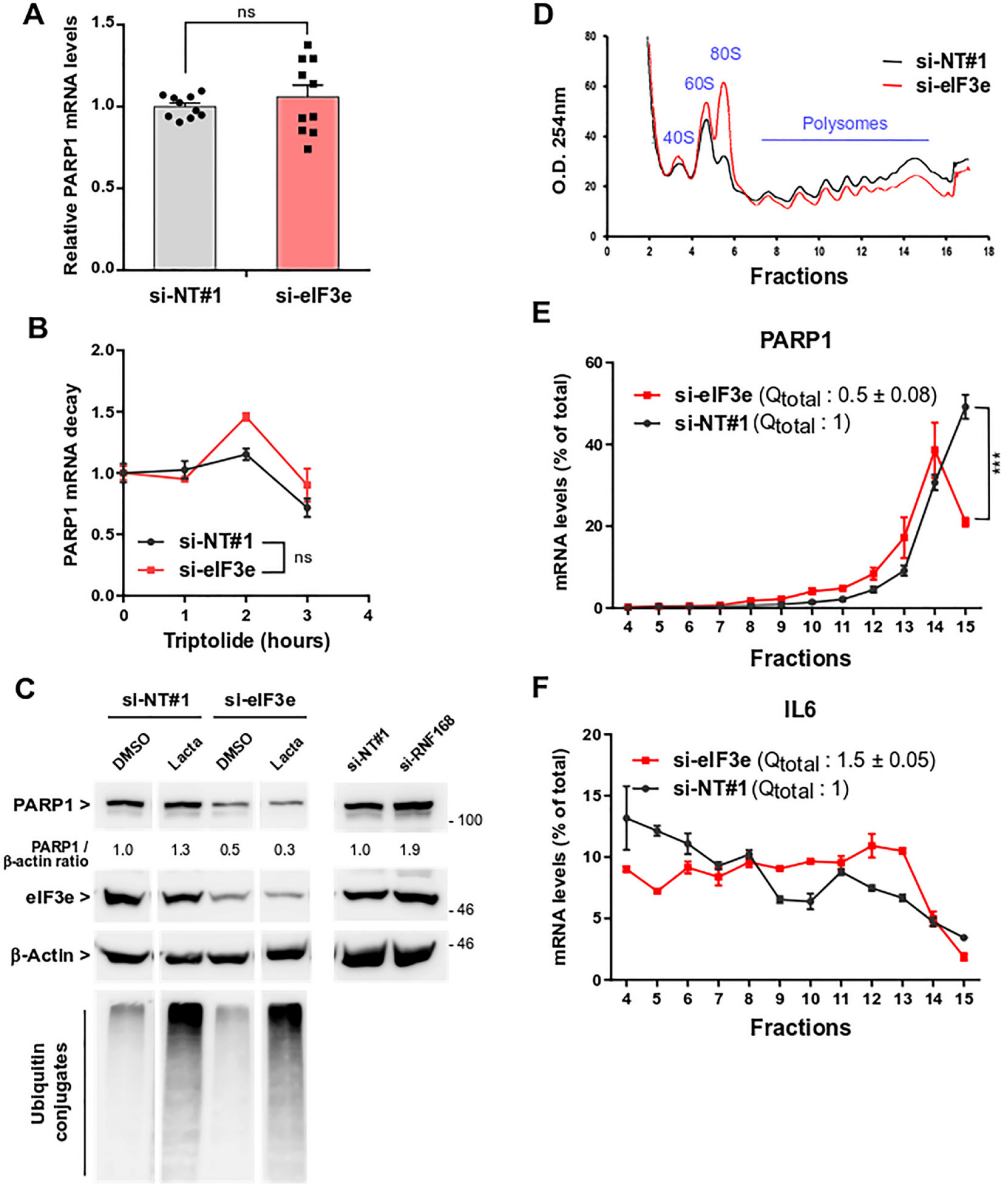
eIF3e is required for efficient PARP1 mRNA translation. (**A**) Quantification of PARP1 mRNAs from MDA-MB-231 cells transfected for 4 days with control or eIF3e siRNAs. PARP1 mRNA levels were measured by reverse transcription and quantitative real-time polymerase chain reaction (qRT-PCR). A value of 1 was assigned to PARP1 level in control cells and measurement in eIF3e-depleted cells was normalized to this value. *n* = 10 from 4 independent experiments. (**B**) PARP1 mRNA decay in MDA-MB-231 cells transfected with siRNAs non-targeting or targeting eIF3e. Four days after transfection, cells were incubated with 20 μM of triptolide to stop *de novo* transcription. Cells were collected at different time points after transcription arrest and PARP1 mRNA levels were measured by qRT-PCR. A value of 1 was assigned to transcript levels of untreated cells, either control- or eIF3e-silenced cells. Results from 2 independent experiments. (**C**) Immunoblots to evaluate PARP1 protein stability in MDA-MB-231 cells transfected with non-targeting siRNA or with siRNAs targeting eIF3e or RNF168 for 4 days. Cells were untreated or treated for the last 8 h with the proteasome inhibitor lactacystin (5 μM), or vehicle only. Data shown for cells treated with si-NT#1 and si-eIF3e correspond to different parts of the same gel. Efficacy of eIF3e depletion and proteasome inhibition were determined by detection of eIF3e and ubiquitin conjugates, respectively. (**D**) UV absorbance profiles of cytoplasmic extracts from MDA-MB-231 cells through a 10% to 50% sucrose gradient. Cells were transfected with siRNAs non-targeting or targeting eIF3e and collected after 3 days. Positions of 40S and 60S ribosomal subunits, 80S monosomes and polysomes are shown. (**E**) Total RNAs were extracted from each gradient fraction and all samples were supplemented with a luciferase RNA spike-in standard to normalize for RNA recovery. The abundance of PARP1 transcript in each gradient fraction was determined using qRT-PCR and is plotted as a percentage of total PARP1 mRNA. A value of 1 was assigned to the total quantity of PARP1 mRNA in control cells. Results from two independent experiments (*n* = 4). (**F**) Quantification of IL6 transcript as in (E). In relevant panels, all error bars represent means ± SEM. All statistical significances were calculated using unpaired *t* test, ^***^ *P* < 0.001, ns, not significant.

To decipher the mechanism of eIF3e-mediated PARP1 regulation, we first measured PARP1 mRNA levels after eIF3e knockdown. No significant changes were observed in MDA-MB-231 cells (Figure 3A) nor in BT-20 cells (Supplementary Figure 3). We also assessed PARP1 mRNA stability since we have previously shown that eIF3e regulates the decay of certain mRNAs [35]. No significant differences were noted in the PARP1 mRNA half-lives upon eIF3e depletion (Figure 3B). We then examined whether silencing eIF3e could alter PARP1 protein stability. One study showed that PARP1 is degraded via the ubiquitin-proteasome system after being ubiquitinated by RNF168 [36]. MDA-MB-231 cells were transfected with siRNAs and treated with the proteasome inhibitor lactacystin. An siRNA against RNF168, used as a positive control, showed a ~2-fold augmentation of PARP1 abundance (Figure 3C). Also, PARP1 levels were increased, albeit to a lesser extent, in control cells treated with the proteasome inhibitor but no stabilization of PARP1was observed in eIF3e-depleted cells.

Next, we reasoned that eIF3e reduction may affect translation of mRNA encoding PARP1. We then wanted to verify this hypothesis by performing polysome profiling followed by PARP1 transcript quantitation. Polysome profiles of eIF3e-depleted MDA-MB-231 cells showed a marked increase in the 80S monosome peak associated with a decrease in polysomes (Figure 3D). Such changes in ribosome profiles are a signature of inhibited translation, suggesting that eIF3e may be required for bulk translation in this cancer cell line. Several studies have reported a slight reduction of global translation upon eIF3e downregulation while other studies did not [24, 25, 37]. The levels of PARP1 transcript engaged in translation were monitored by qRT-PCR and all values were summed to approximate the total quantity of PARP1 mRNA associated with polysomes (Qtotal). Silencing eIF3e caused a 2-fold decrease in the levels of PARP1 transcript recruited for translation (Figure 3E, numbers in parentheses). Furthermore, ~50% of PARP1 mRNA levels in control cells were detected in the heaviest polysome fraction (fraction 15), whereas eIF3e depletion caused a shift towards lighter fractions, with only ~20% of PARP1 mRNA in fraction 15 (Figure 3E). As a comparison, we monitored mRNA encoding the IL6 cytokine that showed a different distribution pattern. Both in control and eIF3e-depleted cells, the IL6 transcript was more regularly distributed across the polysome fractions and was almost undetectable in fraction 15 (Figure 3F), which is consistent with the ~5-fold shorter size of the IL6 open reading frame relative to that of PARP1. We found that eIF3e knockdown induced a 1.5-fold increase in IL6 mRNA engaged in translation (Figure 3F, numbers in parentheses). Also, IL6 transcript was more abundant in heavy fractions (Fractions 9 to 13) of eIF3e-silenced cells compared to control cells. We inferred that eIF3e depletion promotes IL6 mRNA translation. In agreement with this, IL6 is more abundant in eIF3e-deficient cells (see below). Conversely, silencing eIF3e induces a low-efficient translation of PARP1 mRNA, resulting in reduced synthesis of the encoded protein.

### Silencing eIF3e increases replication fork velocity similarly to PARP1 downregulation

**Figure 4:**
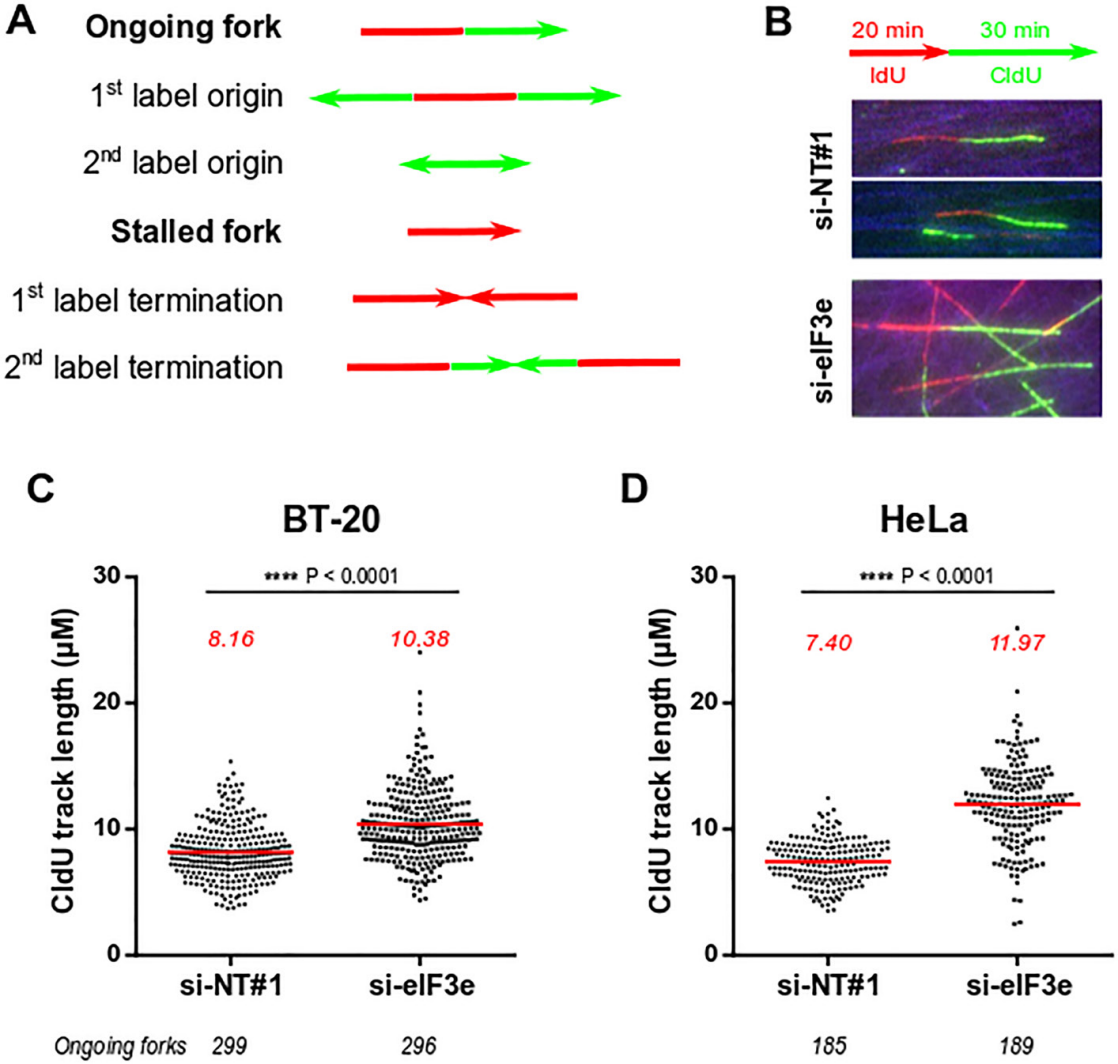
Silencing of eIF3e causes high speed of replication fork. (**A**) Schematic representation of the different replication structures that can be observed by DNA fiber assay and their interpretations. (**B**) Labeling scheme and representative images of ongoing forks from cells transfected with the indicated siRNAs. Cells were sequentially incubated with the two thymidine analogs IdU and CldU, followed by spreading of DNA fibers on glass slides and immunofluorescent staining of DNA segments that have incorporated IdU and/or CldU. (**C**, **D**) Replication fork progression in BT-20 (C) or HeLa (D) cells transfected with siRNAs non-targeting or targeting eIF3e. For a reliable estimation, the length of CldU tracks was measured in a large number of progressing forks indicated below graphs. Lines and numbers in red correspond to values for mean track lengths. Statistical significance was assessed using the Mann-Whitney *U* test.

Besides the impaired synthesis of PAR polymers shown above, we looked at a functional readout for PARP1 defect in eIF3e-depleted cells. One study reveals that downregulating PARP1 expression or activity induces an accelerated progression of DNA replication forks, which triggers a replication stress if above a tolerated threshold [10]. We thus examined whether depleting eIF3e could modify the progression of replication forks. This was monitored using a DNA fiber assay from BT-20 cells treated with siRNAs non-targeting or targeting eIF3e. Image analyses showed different patterns of labelling represented in Figure 4A. Our results did not reveal major changes in their relative proportion between control and eIF3e-depleted cells (Supplementary Figure 4). To evaluate fork progression, we selected DNA segments that have incorporated iodo-deoxyuridine (IdU) and chloro-deoxyuridine (CldU) with no intervening gap (Figure 4B) and we measured the length of ~300 CldU tracks. Our results showed that the CldU track length was increased by ~30% in eIF3e-depleted cells compared to control cells (Figure 4C). To rule out any effect specific to BT-20 cells, we performed the same assay on HeLa cells. We chose this cell line because a previous study reported the effect of PARP1 depletion on fork progression in these cells [10] and we were sure that PARP1 was indeed reduced in eIF3e-silenced HeLa cells (Figure 2D). Our results showed an even stronger effect of eIF3e depletion in these cells. As shown in Figure 4D, the CldU track length was enhanced by ~60% compared to HeLa control cells, which corresponds to an extent similar to that reported for HeLa cells depleted of PARP1 [10]. Together, these findings support the notion that the high replication fork velocity we found in eIF3e-deficient cells may be due to the reduction of PARP1. Furthermore, our data imply that eIF3e-depleted cells could suffer from DNA replication stress, which may drive genomic instability.

### eIF3e depletion promotes senescence and senescence-associated secretory phenotype

**Figure 5:**
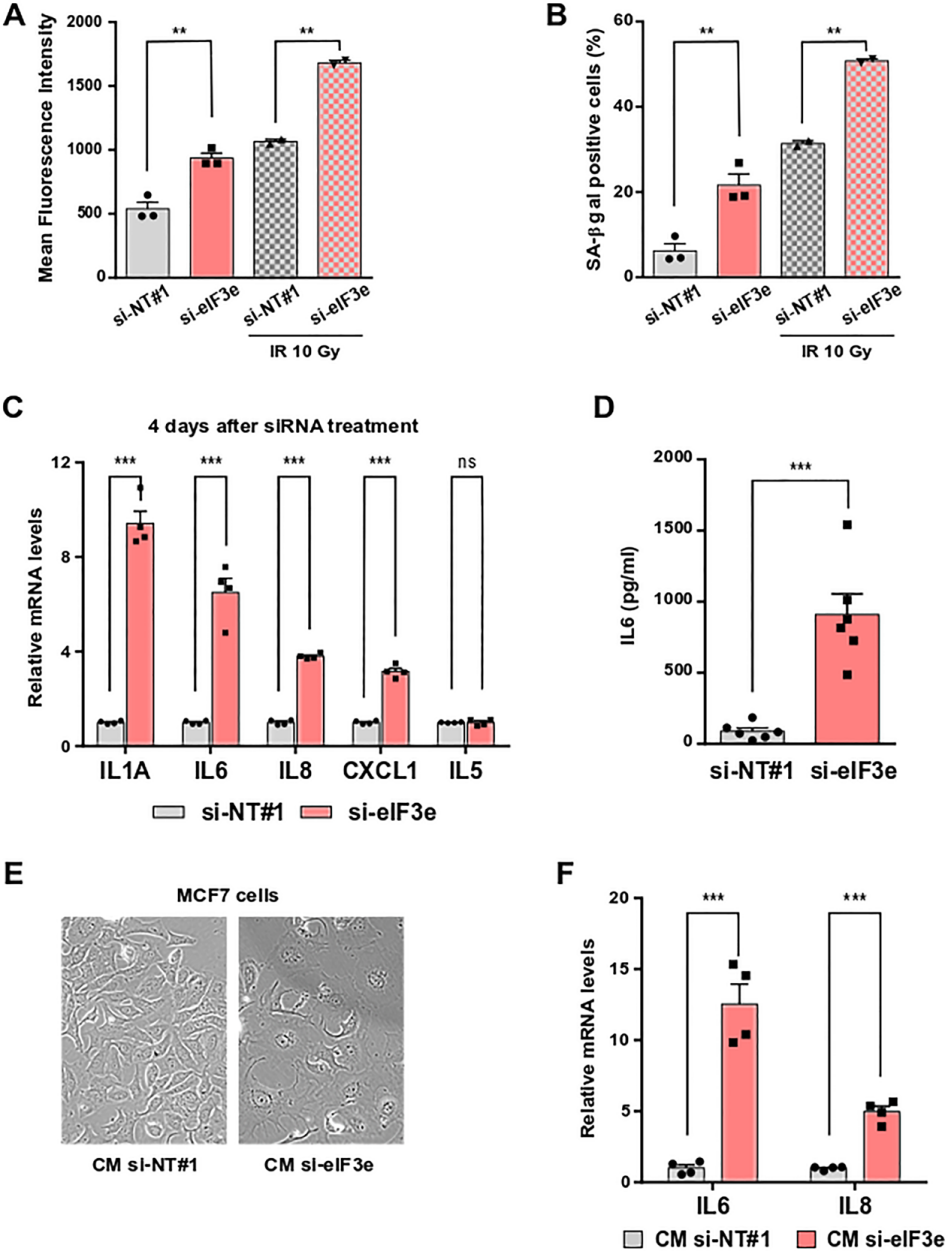
eIF3e depletion promotes senescence and senescence-associated secretory phenotype. (**A**, **B**) MDA-MB-231 cells were transfected with siRNAs non-targeting or targeting eIF3e. Two days later, cells were untreated or X-irradiated (10 Gy) and allowed to grow for 4 days before senescence was assessed using a flow cytometry assay with the substrate C_12_FDG. The bar graph in (A) indicates the mean fluorescence intensity resulting from hydrolysis of C_12_FDG by SA-β-Gal. The bar graph in (B) indicates the percentage of SA-β-Gal positive cells. Results from 3 independent experiments for non-irradiated cells and 2 independent experiments for X-irradiated cells. (**C**) Quantification of mRNAs encoding SASP factors (IL1A, IL6, IL8, and CXCL1) and a non-SASP factor (IL5) from MDA-MB-231 cells transfected for 4 days with control or eIF3e siRNAs. Transcript levels were measured by qRT-PCR and a value of 1 was assigned to mRNA levels of control cells. Results from 4 independent experiments. (**D**) Quantification of IL6 secreted in conditioned media from MDA-MB-231 cells transfected with control or eIF3e siRNAs. Five days after transfection, cells were incubated with a serum-free medium for 24 h before conditioned media were collected and analyzed for IL6 secretion by enzyme-linked immunosorbent assay (ELISA). *n* = 6 from 3 independent experiments. (**E**) MCF7 cells were incubated with conditioned media prepared as described in (D) from MDA-MB-231 cells transfected with siRNAs non-targeting (CM si-NT#1) or targeting eIF3e (CM si-eIF3e). Morphological evaluation was done after 3 days of treatment. Representative phase-contrast images are shown. (**F**) Quantification of IL6 and IL8 transcripts in MCF7 cells treated as in (E). *n* = 4 from two independent experiments. In relevant panels, all error bars represent means ± SEM. All statistical significances were calculated using unpaired *t* test, ^***^ *P* < 0.001, ^**^ *P* < 0.01, ns, not significant.

There is increasing evidence linking replication stress to induction of cellular senescence [1]. Furthermore, reduced expression of PARP1 leads to replication stress [10] and also to induction of cell senescence [8]. Therefore, our results prompted us to investigate whether eIF3e depletion could promote senescence. This was monitored by measuring the activity of senescence-associated β-galactosidase (SA-β-Gal), using a quantitative flow cytometry-based assay [38]. Representative histograms are shown in Supplementary Figure 5A. The relative SA-β-Gal activity was estimated by the mean fluorescence intensity (MFI), which revealed a ~2-fold increase in MFI values in eIF3e-silenced cells compared to control cells (Figure 5A). As positive senescence controls, cells were subjected to X-ray irradiation. Notably, the SA-β-Gal activity of unirradiated eIF3e-depleted cells was similar to that measured in irradiated control siRNA-treated cells (Figure 5A). We then determined the percentage of senescent cells as described in Supplementary Figure 5B, which indicates a 3.5-fold increase in eIF3e-depleted cells (Figure 5B). This senescence promoting effect upon eIF3e knockdown remained effective after irradiation.

One feature of senescent cells is their capacity to secrete a large number of factors collectively termed the SASP [2, 3]. Then, we evaluated the impact of eIF3e reduction on the expression of SASP components in MDA-MB-231 cells. Our qRT-PCR data showed that, compared to a non-targeting control, eIF3e silencing significantly increased mRNA levels of the four SASP factors IL1A, IL6, IL8, and CXCL1, but not of the non-SASP cytokine IL5 (Figure 5C). SASP is a hierarchical process that develops with a gradual increase over several days. In particular, the IL1A cytokine is essential for initiating a signal transduction cascade that ultimately induces the expression and secretion of the IL6 cytokine and IL8 chemokine [39]. Consistent with that, a higher fold change was observed for the IL1A transcript compared to the other SASP mRNAs after eIF3e knockdown for 4 days (Figure 5C). Next, levels of IL6 and IL8 transcripts were measured after siRNA treatment for 6 days. The abundance of both mRNAs was higher in eIF3e-depleted cells at this late time point (~20-fold, Supplementary Figure 6A) compared to an earlier time (Figure 5C). To exclude any off-target effect, a rescue experiment was performed, which showed that expressing an eIF3e cDNA resistant to degradation by the siRNA prevents the SASP monitored through IL1A mRNA levels (Supplementary Figure 6B). Finally, we evaluated the secretion of IL6 using conditioned media prepared from MDA-MB-231 cells treated with siRNAs for 6 days. We found that eIF3e knockdown induced a 10-fold increase in IL6 secretion relative to a control siRNA (Figure 5D). Collectively, these findings suggest that cancer cells deficient for eIF3e prematurely undergo senescence combined with a massive upregulation and secretion of SASP factors.

Several lines of evidence put forward that the SASP promotes tumor progression by maintaining chronic inflammation. Secreted SASP factors, mainly pro-inflammatory cytokines and chemokines, operate as diffusible signals that reinforce and propagate senescence in autocrine and paracrine fashions [40, 41]. To investigate whether the SASP triggered by eIF3e depletion can spread senescence to neighboring cells, we collected conditioned media from MDA-MB-231 cells treated for 6 days with siRNAs control or targeting eIF3e and transferred them to MCF7 cells. We chose these breast cancer cells in particular because MCF7 cells are less aggressive and do not constitutively express IL6 or IL8 [42]. Live cell imaging revealed that, compared to MCF7 cells treated with control conditioned medium, cells treated with conditioned medium from eIF3e-depleted MDA-MB-231 cells exhibited morphological changes characteristic of senescent cells, as enlarged size and flattened appearance (Figure 5E). Additionally, significant levels of IL6 and IL8 transcripts were detected in these cells (Figure 5F). These data indicate that eIF3e deficiency causes propagation of senescence and inflammation to neighboring cells, which might have an impact on cancer progression.

### Induction of SASP in eIF3e-deficient cells occurs through PARP1 inhibition and mTORC1 activation

**Figure 6:**
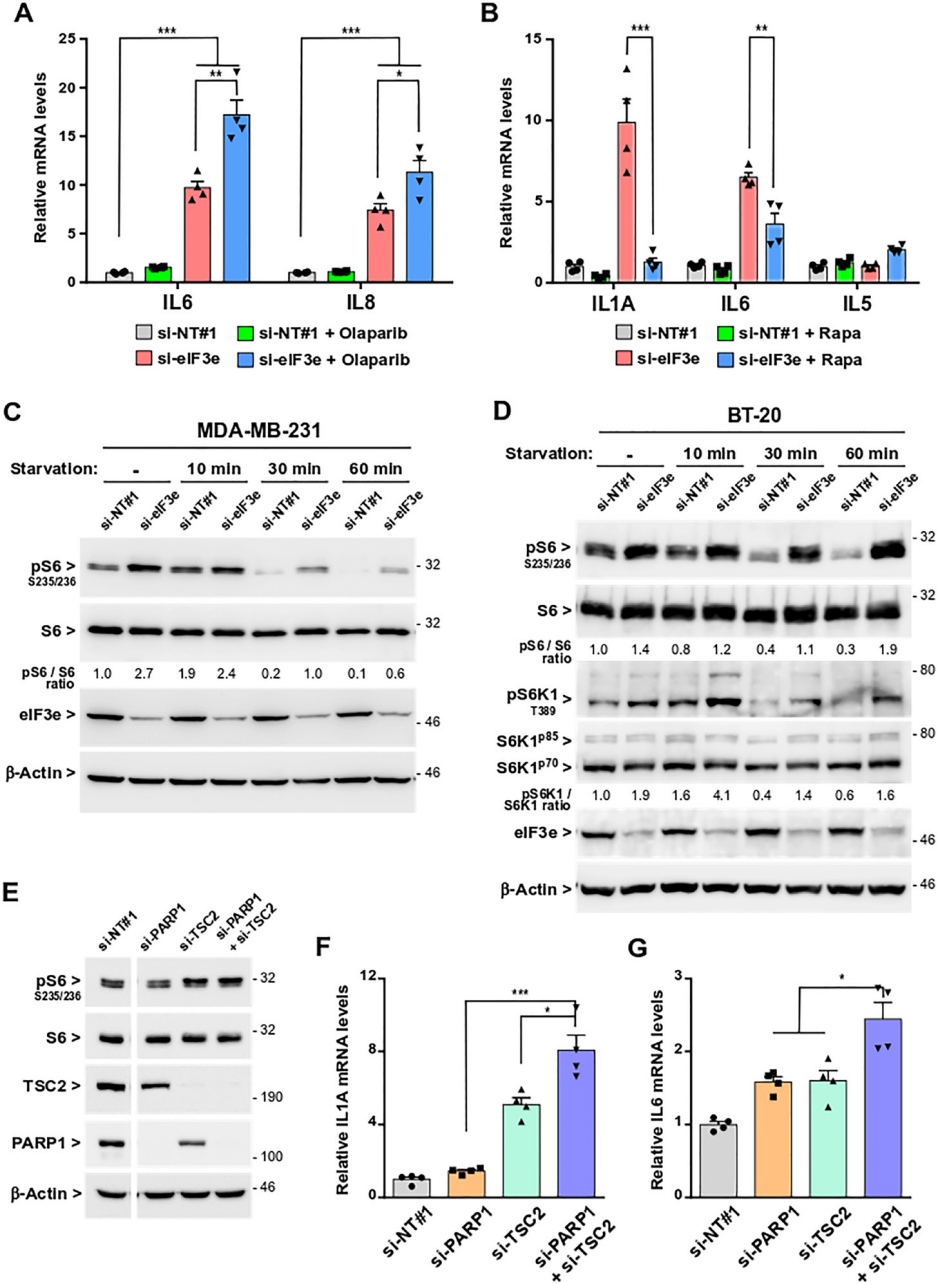
SASP development following eIF3e depletion occurs through PARP1 inhibition and mTORC1 activation. (**A**) Quantification of IL6 and IL8 mRNAs after treatment of eIF3e-silenced cells with olaparib. MDA-MB-231 cells were transfected with siRNAs non-targeting or targeting eIF3e. The following day, cells were untreated or treated with 1 μM of olaparib, and allowed to grow for 4 days before RNA extraction. Transcript levels were measured by qRT-PCR and a value of 1 was assigned to mRNA levels of untreated control cells. *n* = 4 from 2 independent experiments. (**B**) Quantification of mRNAs encoding SASP (IL1A, IL6) and non-SASP (IL5) factors after treatment of eIF3e-silenced cells with rapamycin. MDA-MB-231 cells were transfected as in (A), treated or not with 50 nM of rapamycin 24 h later, and allowed to grow during 3 days before RNA extraction. Transcript levels were measured as in (A). *n* = 4 from 2 independent experiments. (**C**) Detection of S6 phosphorylation level in eIF3e-knockdown cells. MDA-MB-231 cells were transfected with control or eIF3e siRNAs during 3 days before cells were subjected to nutrient starvation for indicated time points. Immunoblots were carried out with the indicated antibodies. Efficiency of eIF3e depletion was determined by detection of eIF3e. (**D**) The phosphorylation levels of S6 and S6K1 were determined in BT-20 cells treated as in (C). Immunoblots were carried out with the indicated antibodies. (**E**) MDA-MB-231 cells were transfected with siRNAs non-targeting or targeting PARP1 and/or TSC2 and harvested 4 days later. Immunoblots were performed using the indicated antibodies. Data shown correspond to two parts of the same gel. Efficiency of RNA interference was determined by detection of TSC2 and PARP1. (**F**, **G**) Quantification of mRNAs encoding IL1A (F) and IL6 (G) in MDA-MB-231 cells transfected with siRNAs non-targeting or targeting PARP1 and/or TSC2 during 4 days. Transcript levels were measured by qRT-PCR and a value of 1 was assigned to mRNA levels of control cells. *n* = 4 from 2 independent experiments. In relevant panels, all error bars represent means ± SEM. All statistical significances were calculated using unpaired *t* test, ^***^ *P* < 0.001, ^**^ *P* < 0.01, ^*^ *P* < 0.05.

Next, we sought to identify signaling networks supporting SASP induction following eIF3e reduction. Previous studies showed that NF-κB and C/EBPβ transcription factors but also signaling through ATM, macro-H2A1, p38MAPK, or mTOR contributes to the SASP [3, 14, 15, 43–45]. Moreover, one study reported that decreased PARP1 expression was sufficient to induce senescence of epithelial cells [8]. First, we tested whether a treatment with the PARPi olaparib could modify SASP magnitude in eIF3e-depleted cells. The idea was that since PARP1 expression is reduced but not completely lost in these cells, inhibition of the residual PARP1 activity may have a synergistical impact on the SASP. Compared to control cells, MDA-MB-231 cells silenced for eIF3e displayed a marked increase in IL6 and IL8 mRNA levels, and this increase was significantly reinforced upon olaparib treatment (Figure 6A). These results indicate that SASP induction in eIF3e-deficient cells is promoted by PARP1 inhibition.

Because inhibition of PARP1 is insufficient for SASP development in control cells, we evaluated whether the SASP induced by eIF3e knockdown could additionally be controlled by mTORC1. The rationale was that this master regulator of protein synthesis interacts with eIF3 [18] and controls the SASP by promoting translation of mRNAs encoding IL1A and MAPKAPK2 [14, 15]. Subsequently, IL1A activates NF-κB which, in turn, stimulates transcription of multiple SASP genes [14, 39] while the MAPKAPK2 kinase indirectly stabilizes several SASP mRNAs by inactivating through phosphorylation the RNA binding protein ZFP36L1, the non-phosphorylated form of which destabilizes many SASP transcripts [15]. To test whether the SASP triggered by eIF3e depletion is linked to mTORC1, siRNA-treated MDA-MB-231 cells were exposed to rapamycin, an mTORC1 inhibitor. Compared to control cells and prior to rapamycin treatment, eIF3e-silenced cells displayed a significant increase in IL1A and IL6 mRNA levels (Figure 6B). Rapamycin treatment was found to blunt the increase of IL6 transcript by ~50% and to almost totally prevent the induction of IL1A transcript in these cells. The levels of mRNA encoding the non-SASP IL5 cytokine were not altered by rapamycin. Together, these results support the notion that mTORC1 activity is required for the SASP in eIF3e-deficient cells.

To further address the link between the SASP and mTORC1 signaling following eIF3e depletion, we assessed mTORC1 activity through the phosphorylation of ribosomal protein S6. Immunoblot analyses showed that, compared to control MDA-MB-231 cells, eIF3e-depleted cells displayed increased levels of S6 phosphorylation, whether these cells were grown under normal nutritional or starved conditions (Figure 6C). Similar observations were obtained using BT-20 cells (Figure 6D). S6 ribosomal protein is not a direct mTORC1 target, but a downstream effector of S6K1, which is itself a mTORC1 substrate. We verified that S6 phosphorylation in eIF3e-depleted cells was mTORC1-dependent by treating cells with rapamycin to inhibit mTORC1 (Supplementary Figure 7A). Although largely used in studying mTORC1 signaling, rapamycin however inhibits more efficiently the S6K1 axis of the mTORC1 signaling compared with the 4E-BP1 axis [46]. For this reason, we assessed the effect of eIF3e depletion directly on phosphorylation of S6K1 and 4E-BP1. Immunoblots confirmed that eIF3e silencing led to increased levels of phosphorylated S6K1 (Figure 6D). Conversely, phosphorylation of 4E-BP1 was unaffected by eIF3e depletion (Supplementary Figure 7B). Finally, a rescue experiment showed that S6 phosphorylation was suppressed by the expression of an eIF3e cDNA resistant to degradation by the siRNA (Supplementary Figure 7C). Together, these findings indicate that the mTORC1-S6K1 signaling axis is aberrantly activated upon eIF3e silencing.

Next, we asked whether combining an overactivation of mTORC1 with a downregulation of PARP1 could recapitulate the SASP features we observed in eIF3e-deficient cells. To overactivate mTORC1, we employed a siRNA targeting the protein tuberous sclerosis complex 2 (TSC2), a negative regulator of mTORC1 [16, 17]. MDA-MB-231 cells were transfected with siRNAs non-targeting or targeting TSC2 and/or PARP1 and mTORC1 activity was assessed through monitoring S6 phosphorylation by immunoblotting. We found that TSC2 depletion led to an increase in S6 phosphorylation (Figure 6E), to an extent comparable to that observed after eIF3e silencing (Figure 6C). Then, we quantified the IL1A and IL6 transcripts to inform about a putative SASP response. Compared to control cells, TSC2-silenced cells displayed a 5-fold increase in IL1A mRNA levels, and this increase was significantly reinforced by combining TSC2 and PARP1 silencing (Figure 6F). This additive effect was also observed for IL6 transcript, although to a lesser extent (Figure 6G). This is most likely because the experimental time point is too short for full SASP development. These results indicate that sustained mTORC1 signaling combined with low PARP1 activity can induce the SASP. Taken together, our data strongly suggest that eIF3e-deficient cells can enter into premature senescence with SASP features because of their decreased expression of PARP1 combined with their aberrant activation of mTORC1.

## DISCUSSION

Based on our previous work establishing a link between eIF3e deficiency and impaired DNA repair by HR [29], we further explored whether eIF3e-dependent HR defects could be exploited in targeted cancer therapy using PARPi. To our surprise, two different breast cancer cell lines deficient for eIF3e did not respond to these drugs. To explain this, we found that PARP1, but not PARP2, was strongly reduced upon eIF3e silencing. In addition to the catalytic inhibition of PARP1/2, all clinical PARPi also trap both proteins onto DNA, with PARP1 being trapped much more efficiently than PARP2 [30]. PARP1 normally dissociates from DNA upon self-PARylation, but by preventing this reaction, PARPi block the enzyme onto DNA and generate a physical obstruction largely responsible for the ensuing cytotoxicity. Therefore, any reduction in PARP1 protein amounts is likely to confer resistance to PARPi, which is what we observed following eIF3e depletion. Hence, one would predict that tumors with low eIF3e levels should be insensitive to PARPi therapy.

Accumulated evidence indicates that PARP1 is the first DNA damage sensor of SSBs and DSBs [6, 7]. Upon binding to DNA breaks, PARP1 extremely rapidly catalyzes extensive PARylation that promotes the recruitment of multiple proteins acting in different aspects of DNA repair. In particular, PARP1 is involved in the early recruitment to DSB sites of the MRN complex and the ATM kinase, which both contain PAR-binding domains [6, 47]. Also, PARP1 PARylates BRCA1 and this modification is important for proper BRCA1 functioning in HR repair [6, 48, 49]. Thus, one may speculate that the eIF3e-mediated reduction in PARP1 levels and PARylation we uncovered in this work is, in part, a causal event to the defects in DNA damage signaling and DSB repair via HR we reported previously [28, 29]. Here, we found that eIF3e depletion causes an acceleration of replication fork speed, which again can be explained by the decreased amounts of PARP1. Indeed, PARP1 is present at DNA replication forks and slows down fork progression through inducing fork reversal or completing full processing of Okazaki fragments [6, 9, 10]. This PARP1-dependent modulation of fork speed represents an important mechanism for stabilizing replication forks. Therefore, our findings that eIF3e deficiency gives rise to DNA repair difficulties and impaired slow-down of replication forks are very likely caused by PARP1 downregulation. As a result, eIF3e-depleted cells may accumulate persistent DNA damage and increased genomic instability.

mTORC1 is a master controller of macromolecule synthesis, best characterized as a positive regulator of protein synthesis [16, 17]. Under nutrient-rich conditions, mTORC1 gets recruited to eIF3 and phosphorylates two key effectors 4E-BP1 and S6K1 [18]. Phosphorylation of 4E-BP1 promotes cap-dependent translation initiation by inducing its release from the initiation factor eIF4E. Phosphorylation of S6K1 controls both translation initiation and elongation. Phosphorylated S6K1 dissociates from eIF3 and, in turn, phosphorylates several substrates, including the ribosomal protein S6. Our results show that eIF3e deficiency constitutively activates the S6K1 branch of mTORC1 signaling. Importantly, S6K1 signaling is required for senescence [50].

Although many questions remain about the complexity of cellular senescence, it has become evident that this state is a cell response to damage occurring in diverse physiological and pathological conditions [1, 4, 5]. Two paramount features characterize senescent cells, namely a stable, rarely reversible, cell cycle arrest associated with a pro-inflammatory secretory phenotype. The general consensus is that senescence initially exerts tumor-suppressive functions, but can provide over-time deleterious effects caused by the SASP-mediated chronic inflammation. Senescent cells accumulate with age and contribute to different age-related diseases, including cancer. Hence, therapeutic opportunities based on specific elimination of senescent cells, otherwise called senotherapies, are being developed to improve healthspan. Also, treatment with mTORC1 inhibitors may have beneficial effects in aging-associated pathologies by suppressing the SASP [14, 15]. Recently, Ogrodnik et al. have proposed a model to explain the vast heterogeneity of senescent phenotypes [51]. Their model unveils that nearly all types of senescence arise from a combination of cell cycle arrest signals together with expansion signals, either growth or proliferation. On this matter, senescence triggered by eIF3e deficiency is consistent with this model. On the one hand, the persistence of DNA damage and replication stress, as a result of decreased PARP1 levels, likely accounts for cell cycle arrest. On the other hand, eIF3e-depleted cells are continuously exposed to expansion signals owing to the unrestrained mTORC1/S6K1signaling. In line with this, we found that combining PARP1 downregulation with mTORC1 constitutive activation induces the SASP in eIF3e-competent cells.

Our study reveals novel molecular alterations in eIF3e-deficient cells that trigger senescence associated with a pro-inflammatory phenotype. Understanding the mechanistic bases by which eIF3e depletion causes mTORC1/S6K1 hyperactivity and a low-efficient translation of PARP1 mRNA will be addressed in future projects. Our findings pave the way for new potential therapeutics to treat eIF3e-deficient breast tumors. While PARPi appear inadequate, such cancers might benefit from senolytic drugs or clinically available mTORC1 inhibitors.

## MATERIALS AND METHODS

### Cell culture and drug treatment

Human MDA-MB-231, MCF7, and HeLa cell lines were obtained from the European Collection of Authenticated Cell Cultures and BT-20 and MCF 10A cell lines from the American Type Culture Collection. Cells were maintained in culture for less than 20 passages after receipt under standard conditions (37°C, 5% CO_2_) and were regularly tested for mycoplasma contamination. All cell lines except MCF 10A cells were maintained in Dulbecco’s Modified Eagle Medium from Gibco, supplemented with 10% fetal bovine serum and antibiotics. MCF 10A cells were cultured using the MEGM^™^ Mammary Epithelial Cell Growth Medium BulletKit^™^ (Lonza). Cells were treated with the following drugs: veliparib (ABT-888; Enzo Life Sciences), olaparib (AZD2281; MedChemExpress), a PARG inhibitor (PDD 00017273; Tocris, bio-techne), H_2_O_2_ (Sigma-Aldrich), rapamycin (Sigma-Aldrich), triptolide (Sigma-Aldrich), lactacystin (Sigma-Aldrich).

### siRNA and plasmid transfection

Cells were transfected with 5 nM of siRNAs using INTERFERin (Polyplus-Transfection). For eIF3e silencing, we purchased the ON-TARGETplus siRNA J-010518-05 (Dharmacon) together with the ON-TARGETplus non-targeting siRNA#1 (D-001810-01) used as control. We purchased the ON-TARGETplus siRNA SMARTpool format for PARP1 silencing (L-006656-03-0005) and TSC2 silencing (L-003029-00-0005). The other siRNA duplexes used in this study were purchased from Sigma-Aldrich. The targeted sequences (sense strand) are: BRCA1, 5′-AGAUAGUUCUACCAGUAAA-3′; RNF168, 5′-GGCGAAGAGCGAUGGAAGA-3′. For silencing and eIF3e add-backs, cells were first transfected with siRNAs using INTERFERin and, the next day, cells were co-transfected with siRNAs and plasmids using jetPRIME (Polyplus-Transfection) according to manufacturer’s instructions. The vector pTL1-HA-eIF3e^R^ was generated using the QuickChange site-directed mutagenesis method (Stratagene) with the following oligos: forward,

5′-ATGACTTCTTCTTGGTCGCATGCTTGGAAGACTTCATTGAAAATGCCCGTCTCTT-3′ and reverse, 5′-CGGGCATTTTCAATGAAGTCTTCCAAGCATGCGACCAAGAAGAAGTCATTCACAA-3′.

### Cell viability assay

Cells were transfected with siRNAs for 24 h and then seeded at 3 × 10^3^ cells per well in a 96-well plate. Cells were allowed to adhere for 8 h and treated with veliparib, olaparib, or vehicle for 4 days. Cell viability was determined by using a CellTiter-Fluor^™^ Cell Viability Assay kit (Promega).

### Conditioned media and ELISA assay

MDA-MB-231 cells were seeded in 10-cm dishes and transfected with siRNAs the next day and 3 days later. Five days after the first siRNA transfection, cells were washed thoroughly and incubated in serum-free medium for 24 h. Conditioned media were collected and clarified by centrifugation at 1000 *g* for 20 min. Cells remaining on dishes were counted for normalization. The volumes of conditioned media were adjusted to match an equivalent number of cells in each sample. The quantifications of secreted IL6 in conditioned media were performed using an ELISA Genie kit (Reagent Genie).

### Measurement of SA-β-Gal activity

The SA-β-Gal activity was determined using a flow cytometry-based assay and the fluorogenic cell permeable substrate C_12_FDG (5-dodecanoylaminofluorescein di-β-D-galactopyranoside; Molecular Probes, ThermoFisher Scientific), as previously described [38]. Briefly, cells were first incubated for 1 h at 37°C in growth medium supplemented with 100 nM of bafilomycin A1 (Sigma-Aldrich) to induce lysosomal alcalinization, followed by an incubation with 33 μM of C_12_FDG for 2 h at 37°C. Cells were trypsinized, washed in PBS, and analyzed immediately using a MACSQuant VYB flow cytometer (Miltenyi Biotec). Flow cytometry data were analyzed using the FlowJo software (TreeStar).

### Immunoblotting

Cell extracts were prepared in Laemmli sample buffer. Protein concentrations were determined using Bradford assays. Proteins were separated on SDS-polyacrylamide gels and transferred to polyvinylidene difluoride membranes. Blots were blocked with 5% dry milk in 0.1% Tween-20 in PBS, incubated with primary antibodies, followed by HRP-conjugated secondary antibodies. The following primary antibodies and dilutions were used: eIF3e (1:1000, C-20, described previously, [52]), β-actin (1:4000, A5441, Sigma-Aldrich), PARP1 [1:1000, 9532, Cell Signaling Technology (CST)], PARP2 (1:1000, NBP2-47337, Novus Biologicals), PAR (1:1000, ALX-804-220-R100, Enzo Life Sciences), Lys48-linked ubiquitin conjugates (1:1000, 05-1307, Millipore), S6 (1:1000, 2217, CST), pS6 (1:2000, 4858, CST), S6K1 (1:1000, 9202, CST), pS6K1 (1:1000, 9234, CST), TSC2 (1:1000, 4308, CST), 4EBP1 (1:1000, 9452, CST), p4EBP1 (1:1000, 9451, CST). Membranes were developed using ECL Prime reagent and scanned with an ImageQuant LAS500 imaging system (GE Healthcare). Bands of interest were quantified with the ImageQuant TL software (GE Healthcare).

### Immunofluorescence and confocal microscopy

Cells were fixed in 4% paraformaldehyde for 10 min, incubated in 100 mmol/L glycine for 10 min, permeabilized with 0.5% Triton X-100 for 5 min, and blocked with 1% BSA for 30 min. Primary antibodies (PARP1 and PAR referenced in the above section) were incubated for 2 h at room temperature and secondary antibodies conjugated with Alexa Fluor 488 or Alexa Fluor 555 (CST) were incubated for 1 h. Nuclei were counterstained with DAPI and slides were mounted in Fluoromount-G medium (Electron Microscopy Sciences). Microscope images were acquired using an LSM 710 confocal microscope (Carl Zeiss) mounted on an Axio Observer Z1 microscope (Carl Zeiss) equipped with a Plan-Apochromat X63/1.4 NA oil-immersion objective. Image acquisition and analysis were performed using LSM ZEN software (Carl Zeiss).

### RNA isolation and quantitative RT-PCR

Total RNAs were extracted with TRIzol (Sigma-Aldrich) and RNA concentrations were measured with a NanoDrop 2000 (Thermo Scientific). qRT-PCR analysis was performed using a QuantiTect SYBR Green RT-PCR kit (Qiagen) and a Rotor-Gene Q cycler (Qiagen), according to cycling conditions specified in the handbook of the kit. The gene-specific primers used are listed below:

PARP1: Forward, GAGTCGGCGATCTTGGACC; Reverse, TGACCCGAGCATTCCTCG

IL1A: Forward, GGTTGAGTTTAAGCCAATCCA; Reverse, TGCTGACCTAGGCTTGATGA

IL6: Forward, AGGAGACTTGCCTGGTGAAA; Reverse, CAGGGGTGGTTATTGCATCT

IL8: Forward, ATGACTTCCAAGCTGGCCGTG; Reverse, TGTGTTGGCGCAGTGTGGTC

CXCL1: Forward, CACCCCAAGAACATCCAAAG; Reverse, TAACTATGGGGGATGCAGGA

IL5: Forward, GGTTTGTTGCAGCCAAAGAT; Reverse, TCTTGGCCCTCATTCTCACT

β-actin: Forward, TTGGGGATCTGTCCACTCC; Reverse, CACACCAGCCACCACTTTC

### Polysome fractionation and RNA preparation

Seventy-two hours after siRNA transfection, cells were washed and harvested by scraping in ice-cold PBS containing 100 μg/mL cycloheximide (Sigma-Aldrich). Cells were then lysed in 0.9 mL of lysis buffer (10 mM Tris-HCl, pH 7.4, 5 mM MgCl_2_, 100 mM KCl, 2 mM DTT, 1% Triton X-100, 100 μg/mL cycloheximide, 1× Protease-Inhibitor Cocktail EDTA-free (Roche), and 450 U RNasin Plus RNase inhibitor (Promega). Lysates were homogenized by gentle pipetting up and down, incubated at 4°C for 10 min, and centrifuged at 2000 *g* for 5 min at 4°C. Supernatants were recovered and further centrifuged at 13 000 *g* for 5 min. Absorbance at 260 nm was measured using an aliquot of the resulting supernatants and 13 optical density (OD) A_260_ units were loaded on top of a 10–50% (w/v) sucrose gradient in 20 mM HEPES-KOH, pH 7.4, 5 mM MgCl_2_, 100 mM KCl, 2 mM DTT, and 100 μg/ mL cycloheximide. Gradients were centrifuged in a SW41 rotor (Beckman) at 35 000 rpm for 2 h 10 min at 4°C. Fifteen fractions were collected with a fraction collector (Brandel Inc) with continuous measurement of the absorbance at 254 nm. RNAs were extracted from each fraction with an equal volume of TRIzol (Sigma-Aldrich) and, prior to RNA precipitation with isopropanol, 5 ng of luciferase spike-in standard RNA (Promega) were added to each sample to serve as a control for the efficiency of RNA isolation and qRT-PCR analysis.

### DNA fiber spreading assay

Seventy-two hours after siRNA transfection, cells were sequentially labelled with 20 μM 5-Iodo-2′-deoxyuridine (IdU; Sigma-Aldrich) for 20 min and then with 200 μM 5-Chloro-2′-deoxyuridine (CldU; Sigma-Aldrich) for 30 min. DNA fibers were prepared from 1000 cells placed onto a glass slide and lysed with spreading buffer (200 mM Tris-HCl pH 7.5, 50 mM EDTA, 0.5% SDS) by gently stirring with a pipette tip. The slides were tilted slightly and the drops were allowed to run down the slides slowly, then air-dried, fixed in 3:1 methanol:acetic acid for 10 min, and allowed to dry. DNA fibers were denatured with 2.5M HCl for 1 h, washed with PBS, and blocked in PBS with 1% BSA and 0.1% Tween 20 for 1 h. The newly replicated IdU and CldU tracts and DNA fibers were revealed with mouse anti-BrdU (1:100, clone B44, 347580, BD Biosciences), rat anti-BrdU (1:100, clone BU1/75, ab6326, Abcam), and mouse anti-ssDNA antibodies (1:25, Developmental Studies Hybridoma Bank), respectively. These primary antibodies diluted in blocking buffer were incubated at 37°C for 45 min. Slides were washed in blocking buffer and incubated at 37°C for 20 min with the following secondary antibodies: for IdU, goat anti-mouse IgG1 conjugated to Alexa Fluor 546 (1:100, A21123, ThermoFisher Scientific); for CldU, chicken anti-rat IgG conjugated to Alexa Fluor 488 (1:100, A21470, ThermoFisher Scientific); for DNA, goat anti-mouse IgG2a conjugated to Alexa Fluor 647 (1:50, A21241, ThermoFisher Scientific). Images were acquired with a Zeiss Axio Imager Z1 microscope equipped with a CoolSNAP camera (Photometrics) and a X63/1.4 NA oil-immersion objective. Image processing was performed with ImageJ software and statistical analysis was done using GraphPad Prism using two-sided Mann-Whitney *U* test.

### Statistical analysis

Graphs and statistical analysis were performed using the GraphPad Prism software (version 6). Data are expressed as means ± SEM. Comparisons between two groups were assessed by unpaired, two-tailed *t* test or two-sided Mann-Whitney *U* test. *P* < 0.05 was considered statistically significant. Statistical details and significance levels can be found in the figure legends.

## SUPPLEMENTARY MATERIALS



## References

[1] Gorgoulis V , Adams PD , Alimonti A , Bennett DC , Bischof O , Bishop C , Campisi J , Collado M , Evangelou K , Ferbeyre G , Gil J , Hara E , Krizhanovsky V , et al. Cellular Senescence: Defining a Path Forward. Cell. 2019; 179:813–27. 10.1016/j.cell.2019.10.005. 31675495

[2] Coppé JP , Patil CK , Rodier F , Sun Y , Muñoz DP , Goldstein J , Nelson PS , Desprez PY , Campisi J . Senescence-associated secretory phenotypes reveal cell-nonautonomous functions of oncogenic RAS and the p53 tumor suppressor. PLoS Biol. 2008; 6:2853–68. 10.1371/journal.pbio.0060301. 19053174 PMC2592359

[3] Kuilman T , Michaloglou C , Vredeveld LCW , Douma S , van Doorn R , Desmet CJ , Aarden LA , Mooi WJ , Peeper DS . Oncogene-induced senescence relayed by an interleukin-dependent inflammatory network. Cell. 2008; 133:1019–31. 10.1016/j.cell.2008.03.039. 18555778

[4] Milanovic M , Fan DNY , Belenki D , Däbritz JHM , Zhao Z , Yu Y , Dörr JR , Dimitrova L , Lenze D , Monteiro Barbosa IA , Mendoza-Parra MA , Kanashova T , Metzner M , et al. Senescence-associated reprogramming promotes cancer stemness. Nature. 2018; 553:96–100. 10.1038/nature25167. 29258294

[5] Pluquet O , Abbadie C , Coqueret O . Connecting cancer relapse with senescence. Cancer Lett. 2019; 463:50–8. 10.1016/j.canlet.2019.08.004. 31404612

[6] Ray Chaudhuri A , Nussenzweig A . The multifaceted roles of PARP1 in DNA repair and chromatin remodelling. Nat Rev Mol Cell Biol. 2017; 18:610–21. 10.1038/nrm.2017.53. 28676700 PMC6591728

[7] Martin-Hernandez K , Rodriguez-Vargas JM , Schreiber V , Dantzer F . Expanding functions of ADP-ribosylation in the maintenance of genome integrity. Semin Cell Dev Biol. 2017; 63:92–101. 10.1016/j.semcdb.2016.09.009. 27670719

[8] Nassour J , Martien S , Martin N , Deruy E , Tomellini E , Malaquin N , Bouali F , Sabatier L , Wernert N , Pinte S , Gilson E , Pourtier A , Pluquet O , Abbadie C . Defective DNA single-strand break repair is responsible for senescence and neoplastic escape of epithelial cells. Nat Commun. 2016; 7:10399. 10.1038/ncomms10399. 26822533 PMC4740115

[9] Hanzlikova H , Kalasova I , Demin AA , Pennicott LE , Cihlarova Z , Caldecott KW . The Importance of Poly(ADP-Ribose) Polymerase as a Sensor of Unligated Okazaki Fragments during DNA Replication. Mol Cell. 2018; 71:319–331.e3. 10.1016/j.molcel.2018.06.004. 29983321 PMC6060609

[10] Maya-Mendoza A , Moudry P , Merchut-Maya JM , Lee M , Strauss R , Bartek J . High speed of fork progression induces DNA replication stress and genomic instability. Nature. 2018; 559:279–84. 10.1038/s41586-018-0261-5. 29950726

[11] Bryant HE , Schultz N , Thomas HD , Parker KM , Flower D , Lopez E , Kyle S , Meuth M , Curtin NJ , Helleday T . Specific killing of BRCA2-deficient tumours with inhibitors of poly(ADP-ribose) polymerase. Nature. 2005; 434:913–7. 10.1038/nature03443. 15829966

[12] Farmer H , McCabe N , Lord CJ , Tutt ANJ , Johnson DA , Richardson TB , Santarosa M , Dillon KJ , Hickson I , Knights C , Martin NMB , Jackson SP , Smith GCM , Ashworth A . Targeting the DNA repair defect in BRCA mutant cells as a therapeutic strategy. Nature. 2005; 434:917–21. 10.1038/nature03445. 15829967

[13] Pommier Y , O'Connor MJ , de Bono J . Laying a trap to kill cancer cells: PARP inhibitors and their mechanisms of action. Sci Transl Med. 2016; 8:362ps17. 10.1126/scitranslmed.aaf9246. 27797957

[14] Laberge RM , Sun Y , Orjalo AV , Patil CK , Freund A , Zhou L , Curran SC , Davalos AR , Wilson-Edell KA , Liu S , Limbad C , Demaria M , Li P , et al. MTOR regulates the pro-tumorigenic senescence-associated secretory phenotype by promoting IL1A translation. Nat Cell Biol. 2015; 17:1049–61. 10.1038/ncb3195. 26147250 PMC4691706

[15] Herranz N , Gallage S , Mellone M , Wuestefeld T , Klotz S , Hanley CJ , Raguz S , Acosta JC , Innes AJ , Banito A , Georgilis A , Montoya A , Wolter K , et al. mTOR regulates MAPKAPK2 translation to control the senescence-associated secretory phenotype. Nat Cell Biol. 2015; 17:1205–17. 10.1038/ncb3225. 26280535 PMC4589897

[16] Saxton RA , Sabatini DM . mTOR Signaling in Growth, Metabolism, and Disease. Cell. 2017; 168:960–76. 10.1016/j.cell.2017.02.004. 28283069 PMC5394987

[17] Mossmann D , Park S , Hall MN . mTOR signalling and cellular metabolism are mutual determinants in cancer. Nat Rev Cancer. 2018; 18:744–57. 10.1038/s41568-018-0074-8. 30425336

[18] Holz MK , Ballif BA , Gygi SP , Blenis J . mTOR and S6K1 mediate assembly of the translation preinitiation complex through dynamic protein interchange and ordered phosphorylation events. Cell. 2005; 123:569–80. 10.1016/j.cell.2005.10.024. 16286006

[19] des Georges A , Dhote V , Kuhn L , Hellen CUT , Pestova TV , Frank J , Hashem Y . Structure of mammalian eIF3 in the context of the 43S preinitiation complex. Nature. 2015; 525:491–5. 10.1038/nature14891. 26344199 PMC4719162

[20] Smith MD , Arake-Tacca L , Nitido A , Montabana E , Park A , Cate JH . Assembly of eIF3 Mediated by Mutually Dependent Subunit Insertion. Structure. 2016; 24:886–96. 10.1016/j.str.2016.02.024. 27210288 PMC4938246

[21] Cate JHD . Human eIF3: from “blobology” to biological insight. Philos Trans R Soc Lond B Biol Sci. 2017; 372:20160176. 10.1098/rstb.2016.0176. 28138064 PMC5311922

[22] Marchetti A , Buttitta F , Pellegrini S , Bertacca G , Callahan R . Reduced expression of INT-6/eIF3-p48 in human tumors. Int J Oncol. 2001; 18:175–9. 10.3892/ijo.18.1.175. 11115556

[23] Umar A , Kang H , Timmermans AM , Look MP , Meijer-van Gelder ME , den Bakker MA , Jaitly N , Martens JWM , Luider TM , Foekens JA , Pasa-Tolić L . Identification of a putative protein profile associated with tamoxifen therapy resistance in breast cancer. Mol Cell Proteomics. 2009; 8:1278–94. 10.1074/mcp.M800493-MCP200. 19329653 PMC2690491

[24] Suo J , Snider SJ , Mills GB , Creighton CJ , Chen AC , Schiff R , Lloyd RE , Chang EC . Int6 regulates both proteasomal degradation and translation initiation and is critical for proper formation of acini by human mammary epithelium. Oncogene. 2011; 30:724–36. 10.1038/onc.2010.445. 20890303 PMC3017639

[25] Gillis LD , Lewis SM . Decreased eIF3e/Int6 expression causes epithelial-to-mesenchymal transition in breast epithelial cells. Oncogene. 2013; 32:3598–605. 10.1038/onc.2012.371. 22907435

[26] Suo J , Medina D , Herrera S , Zheng ZY , Jin L , Chamness GC , Contreras A , Gutierrez C , Hilsenbeck S , Umar A , Foekens JA , Hanash S , Schiff R , et al. Int6 reduction activates stromal fibroblasts to enhance transforming activity in breast epithelial cells. Cell Biosci. 2015; 5:10. 10.1186/s13578-015-0001-6. 25774287 PMC4359526

[27] Sesen J , Casaos J , Scotland SJ , Seva C , Eisinger-Mathason TSK , Skuli N . The Bad, the Good and eIF3e/INT6. Front Biosci (Landmark Ed). 2017; 22:1–20. 10.2741/4469. 27814599

[28] Morris C , Tomimatsu N , Richard DJ , Cluet D , Burma S , Khanna KK , Jalinot P . INT6/EIF3E interacts with ATM and is required for proper execution of the DNA damage response in human cells. Cancer Res. 2012; 72:2006–16. 10.1158/0008-5472.CAN-11-2562. 22508697 PMC3335344

[29] Morris C , Tomimatsu N , Burma S , Jalinot P . INT6/EIF3E Controls the RNF8-Dependent Ubiquitylation Pathway and Facilitates DNA Double-Strand Break Repair in Human Cells. Cancer Res. 2016; 76:6054–65. 10.1158/0008-5472.CAN-16-0723. 27550454 PMC5065779

[30] Murai J , Huang SY , Das BB , Renaud A , Zhang Y , Doroshow JH , Ji J , Takeda S , Pommier Y . Trapping of PARP1 and PARP2 by Clinical PARP Inhibitors. Cancer Res. 2012; 72:5588–99. 10.1158/0008-5472.CAN-12-2753. 23118055 PMC3528345

[31] D’Andrea AD . Mechanisms of PARP inhibitor sensitivity and resistance. DNA Repair (Amst). 2018; 71:172–6. 10.1016/j.dnarep.2018.08.021. 30177437

[32] Pettitt SJ , Rehman FL , Bajrami I , Brough R , Wallberg F , Kozarewa I , Fenwick K , Assiotis I , Chen L , Campbell J , Lord CJ , Ashworth A . A genetic screen using the PiggyBac transposon in haploid cells identifies Parp1 as a mediator of olaparib toxicity. PLoS One. 2013; 8:e61520. 10.1371/journal.pone.0061520. 23634208 PMC3636235

[33] Bajrami I , Frankum JR , Konde A , Miller RE , Rehman FL , Brough R , Campbell J , Sims D , Rafiq R , Hooper S , Chen L , Kozarewa I , Assiotis I , et al. Genome-wide profiling of genetic synthetic lethality identifies CDK12 as a novel determinant of PARP1/2 inhibitor sensitivity. Cancer Res. 2014; 74:287–97. 10.1158/0008-5472.CAN-13-2541. 24240700 PMC4886090

[34] Johansson HJ , Socciarelli F , Vacanti NM , Haugen MH , Zhu Y , Siavelis I , Fernandez-Woodbridge A , Aure MR , Sennblad B , Vesterlund M , Branca RM , Orre LM , Huss M , et al, and Consortia Oslo Breast Cancer Research Consortium (OSBREAC). Breast cancer quantitative proteome and proteogenomic landscape. Nat Commun. 2019; 10:1600. 10.1038/s41467-019-09018-y. 30962452 PMC6453966

[35] Morris C , Wittmann J , Jaeck HM , Jalinot P . Human INT6/eIF3e is required for nonsense-mediated mRNA decay. EMBO Rep. 2007; 8:596–602. 10.1038/sj.embor.7400955. 17468741 PMC2002529

[36] Kim JJ , Lee SY , Kim S , Chung JM , Kwon M , Yoon JH , Park S , Hwang Y , Park D , Lee JS , Kang HC . A Novel Reciprocal Crosstalk between RNF168 and PARP1 to Regulate DNA Repair Processes. Mol Cells. 2018; 41:799–807. 10.14348/molcells.2018.0078. 30037213 PMC6125419

[37] Grzmil M , Rzymski T , Milani M , Harris AL , Capper RG , Saunders NJ , Salhan A , Ragoussis J , Norbury CJ . An oncogenic role of eIF3e/INT6 in human breast cancer. Oncogene. 2010; 29:4080–9. 10.1038/onc.2010.152. 20453879

[38] Cahu J , Sola B . A sensitive method to quantify senescent cancer cells. J Vis Exp. 2013; 78:50494. 10.3791/50494. 23963434 PMC3846801

[39] Orjalo AV , Bhaumik D , Gengler BK , Scott GK , Campisi J . Cell surface-bound IL-1alpha is an upstream regulator of the senescence-associated IL-6/IL-8 cytokine network. Proc Natl Acad Sci U S A. 2009; 106:17031–6. 10.1073/pnas.0905299106. 19805069 PMC2761322

[40] Acosta JC , O’Loghlen A , Banito A , Guijarro MV , Augert A , Raguz S , Fumagalli M , Da Costa M , Brown C , Popov N , Takatsu Y , Melamed J , d’Adda di Fagagna F , et al. Chemokine signaling via the CXCR2 receptor reinforces senescence. Cell. 2008; 133:1006–18. 10.1016/j.cell.2008.03.038. 18555777

[41] Acosta JC , Banito A , Wuestefeld T , Georgilis A , Janich P , Morton JP , Athineos D , Kang TW , Lasitschka F , Andrulis M , Pascual G , Morris KJ , Khan S , et al. A complex secretory program orchestrated by the inflammasome controls paracrine senescence. Nat Cell Biol. 2013; 15:978–90. 10.1038/ncb2784. 23770676 PMC3732483

[42] Ortiz-Montero P , Londoño-Vallejo A , Vernot JP . Senescence-associated IL-6 and IL-8 cytokines induce a self- and cross-reinforced senescence/inflammatory milieu strengthening tumorigenic capabilities in the MCF-7 breast cancer cell line. Cell Commun Signal. 2017; 15:17. 10.1186/s12964-017-0172-3. 28472950 PMC5418812

[43] Rodier F , Coppé JP , Patil CK , Hoeijmakers WAM , Muñoz DP , Raza SR , Freund A , Campeau E , Davalos AR , Campisi J . Persistent DNA damage signalling triggers senescence-associated inflammatory cytokine secretion. Nat Cell Biol. 2009; 11:973–9. 10.1038/ncb1909. 19597488 PMC2743561

[44] Chen H , Ruiz PD , McKimpson WM , Novikov L , Kitsis RN , Gamble MJ . MacroH2A1 and ATM Play Opposing Roles in Paracrine Senescence and the Senescence-Associated Secretory Phenotype. Mol Cell. 2015; 59:719–31. 10.1016/j.molcel.2015.07.011. 26300260 PMC4548812

[45] Freund A , Patil CK , Campisi J . p38MAPK is a novel DNA damage response-independent regulator of the senescence-associated secretory phenotype. EMBO J. 2011; 30:1536–48. 10.1038/emboj.2011.69. 21399611 PMC3102277

[46] Thoreen CC , Kang SA , Chang JW , Liu Q , Zhang J , Gao Y , Reichling LJ , Sim T , Sabatini DM , Gray NS . An ATP-competitive mammalian target of rapamycin inhibitor reveals rapamycin-resistant functions of mTORC1. J Biol Chem. 2009; 284:8023–32. 10.1074/jbc.M900301200. 19150980 PMC2658096

[47] Haince JF , Kozlov S , Dawson VL , Dawson TM , Hendzel MJ , Lavin MF , Poirier GG . Ataxia telangiectasia mutated (ATM) signaling network is modulated by a novel poly(ADP-ribose)-dependent pathway in the early response to DNA-damaging agents. J Biol Chem. 2007; 282:16441–53. 10.1074/jbc.M608406200. 17428792

[48] Li M , Yu X . Function of BRCA1 in the DNA damage response is mediated by ADP-ribosylation. Cancer Cell. 2013; 23:693–704. 10.1016/j.ccr.2013.03.025. 23680151 PMC3759356

[49] Hu Y , Petit SA , Ficarro SB , Toomire KJ , Xie A , Lim E , Cao SA , Park E , Eck MJ , Scully R , Brown M , Marto JA , Livingston DM . PARP1-driven poly-ADP-ribosylation regulates BRCA1 function in homologous recombination-mediated DNA repair. Cancer Discov. 2014; 4:1430–47. 10.1158/2159-8290.CD-13-0891. 25252691 PMC4258125

[50] Barilari M , Bonfils G , Treins C , Koka V , De Villeneuve D , Fabrega S , Pende M . ZRF1 is a novel S6 kinase substrate that drives the senescence programme. EMBO J. 2017; 36:736–50. 10.15252/embj.201694966. 28242756 PMC5350561

[51] Ogrodnik M , Salmonowicz H , Jurk D , Passos JF . Expansion and Cell-Cycle Arrest: Common Denominators of Cellular Senescence. Trends Biochem Sci. 2019; 44:996–1008. 10.1016/j.tibs.2019.06.011. 31345557

[52] Morris-Desbois C , Bochard V , Reynaud C , Jalinot P . Interaction between the Ret finger protein and the int-6 gene product and colocalisation into nuclear bodies. J Cell Sci. 1999; 112:3331–42. 10504338 10.1242/jcs.112.19.3331

