# Supplementary material for: Decreased expression of the translation factor eIF3e induces senescence in breast cancer cells via suppression of PARP1 and activation of mTORC1
Source: Oncotarget. 2021 Mar 30;12(7):649–64. doi: 10.18632/oncotarget.27923 (PMC8021025; doi:10.18632/oncotarget.27923)
Supplement: Supplementary file 1 [file oncotarget-12-649-s001.pdf]

Decreased expression of the translation factor eIF3e induces senescence in breast cancer cells via suppression of PARP1 and activation of mTORC1

SUPPLEMENTARY MATERIALS

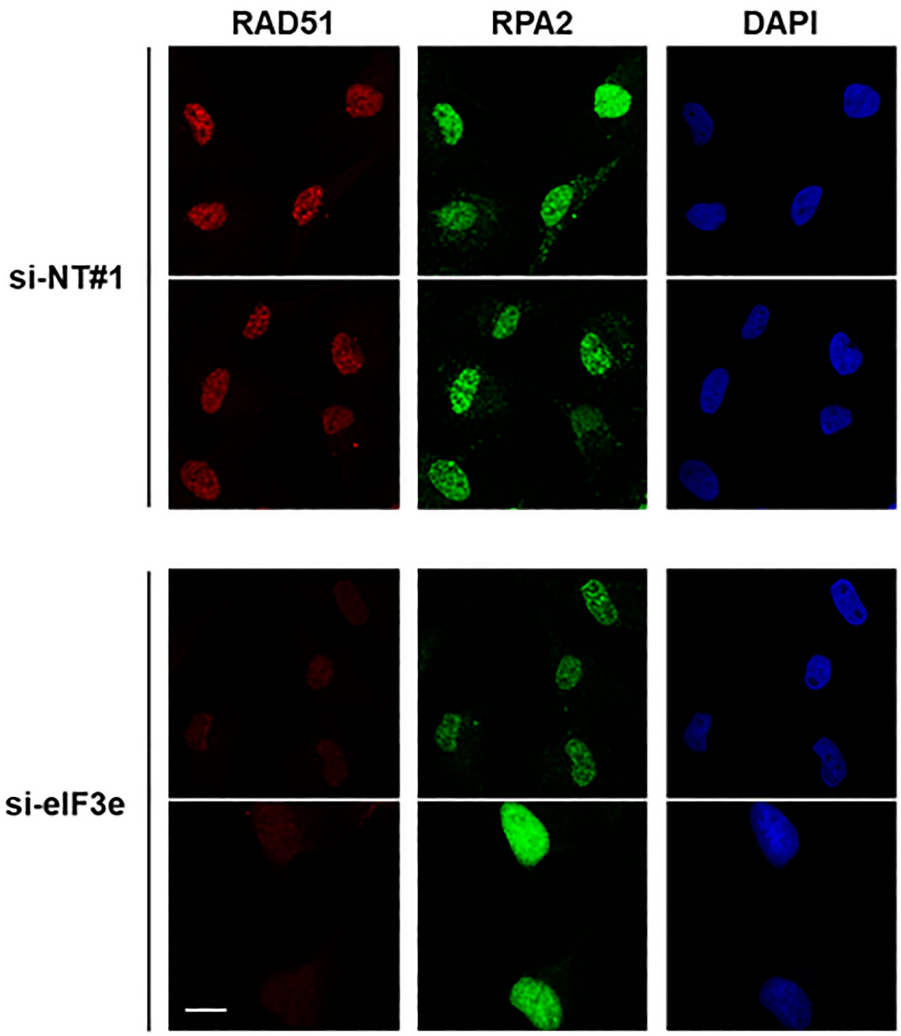

Supplementary Figure 1: HR-mediated DNA repair is impaired in breast cancer BT-20 cells depleted for eIF3e. BT-20 cells were transfected with control or eIF3e siRNAs for 72 hours, X-ray irradiated (6 Gy), immunostained 2 hours later with antibodies to RAD51 and RPA2, and nuclei were stained with DAPI. Representative confocal images show that the RAD51 recombinase from the HR pathway is not recruited at sites of DNA damage in eIF3e-depleted cells, in contrast to the RPA protein. Scale bar, 10 µm.

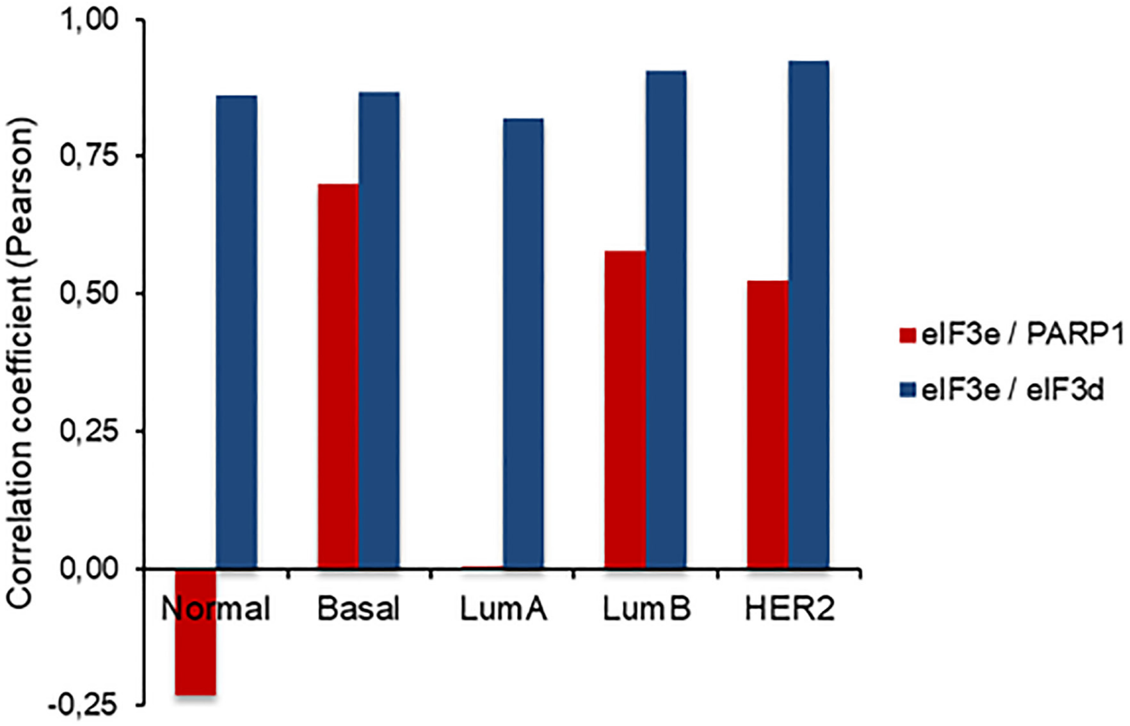

Supplementary Figure 2: Correlation analysis of eIF3e and PARP1 protein abundances using the online breast cancer proteome resource www.breastcancerlandscape.org created by Johansson et al. (Nature Communications 2019; 10:1600). This resource provides quantitative proteomic data from 45 breast cancers classified into five tumoral subtypes (normal-like, basal-like, luminal A & B, and HER2). Interestingly, a significant positive correlation was found for the abundances of eIF3e and PARP1 in the basal-like tumoral subtype, which grossly corresponds to the aggressive triple-negative tumor group. As a positive control, correlation analysis was performed between the protein levels of eIF3e and eIF3d. As expected, a high correlation coefficient was obtained for the two subunits of the eIF3 complex in all five tumor subtypes.

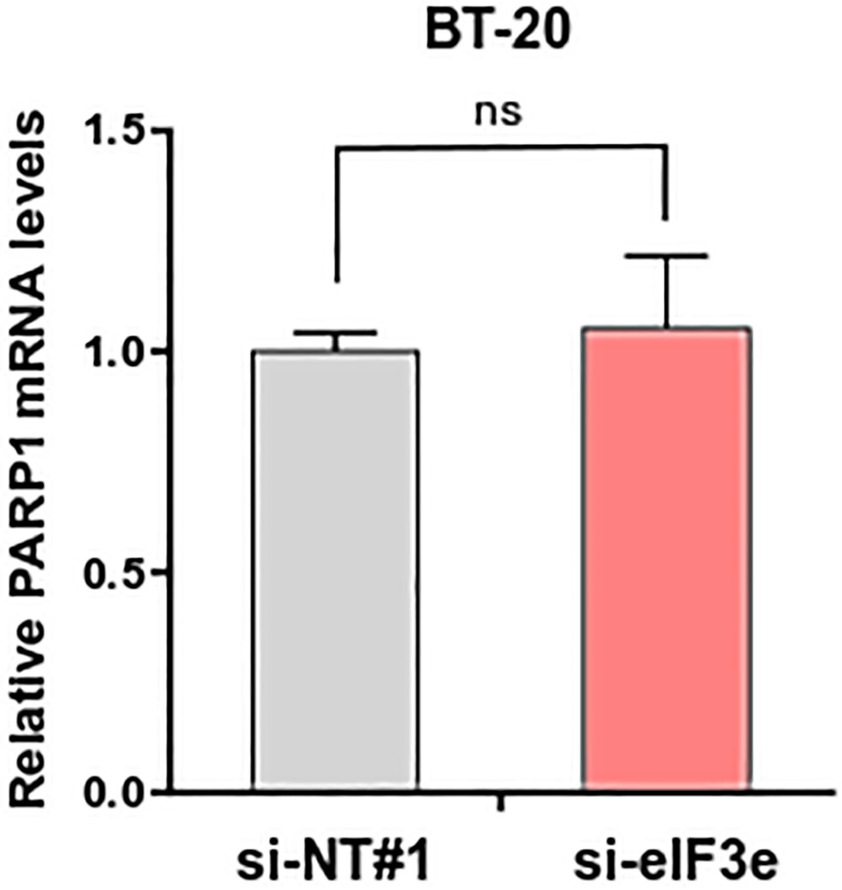

Supplementary Figure 3: PARP1 mRNA levels do not change in BT-20 breast cancer cells depleted of eIF3e. Quantification of PARP1 mRNAs from BT-20 cells transfected for 4 days with siRNAs non-targeting or targeting eIF3e. PARP1 mRNA levels were measured by qRT-PCR. A value of 1 was assigned to PARP1 level in control cells and measurement in eIF3e-depleted cells was normalized to this value. Error bars, means ± SEM. ns, not significant, by unpaired two-tailed t test. Results from 2 independent experiments.

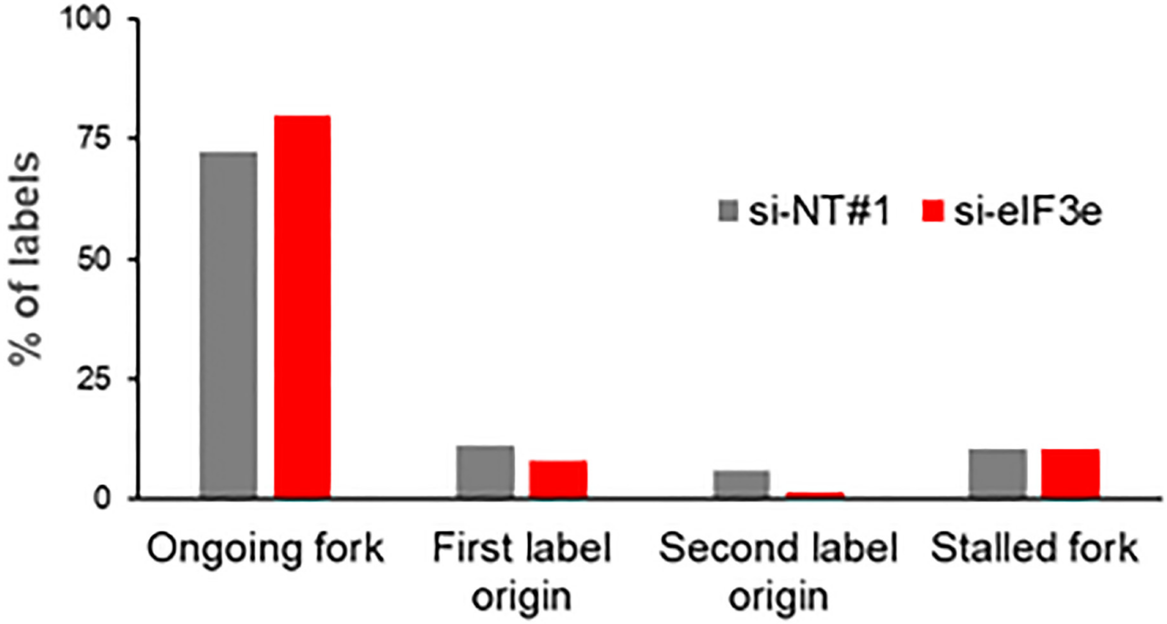

Supplementary Figure 4: Relative proportion of the different replication structures observed in DNA fiber spreading assays. DNA fibers were prepared from BT-20 cells transfected with siRNAs non-targeting or targeting eIF3e for 3 days. Graph indicates the relative proportion of ongoing forks, first- and second-labelled origin forks, and stalled forks.

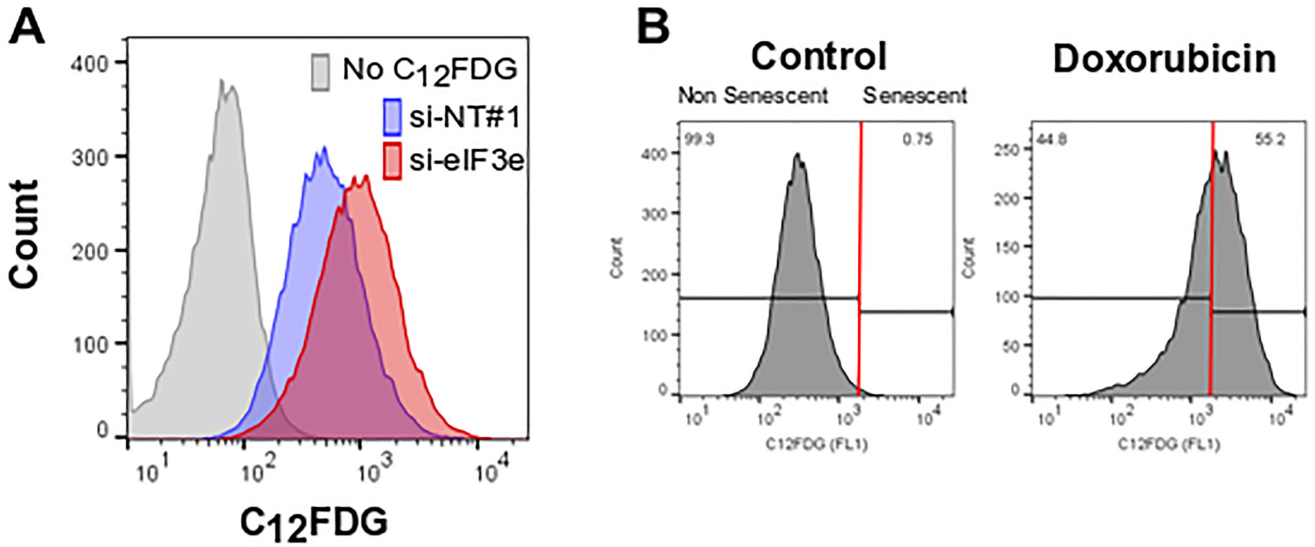

Supplementary Figure 5: Flow cytometric analysis of senescence using C_{12}FDG as a substrate of SA-β-Gal. (**A**) MDA-MB-231 cells were transfected with siRNAs non-targeting or targeting eIF3e. Six days later, cells were first treated with bafilomycin A1 (100 nM for 1 h), to neutralize the acidic pH of lysosomes, and then incubated with C_{12}FDG (33 µM for 2 h) before analysis by flow cytometry. Shown are representative histograms of control cells and eIF3e-silenced cells, where the *x* axis indicates the fluorescence of C_{12}FDG and the y axis indicates the number of events. Cells not treated with C_{12}FDG were used as a negative control. (**B**) To correctly quantify the percentage of senescent cells following eIF3e knockdown, we processed in parallel parental MDA-MB-231 cells that were left untreated or treated with doxorubicin to induce senescence. The corresponding C_{12}FDG histograms were divided in two parts by setting up a boundary (in red) between weakly fluorescent cells (non-senescent) and brightly fluorescent cells (senescent).

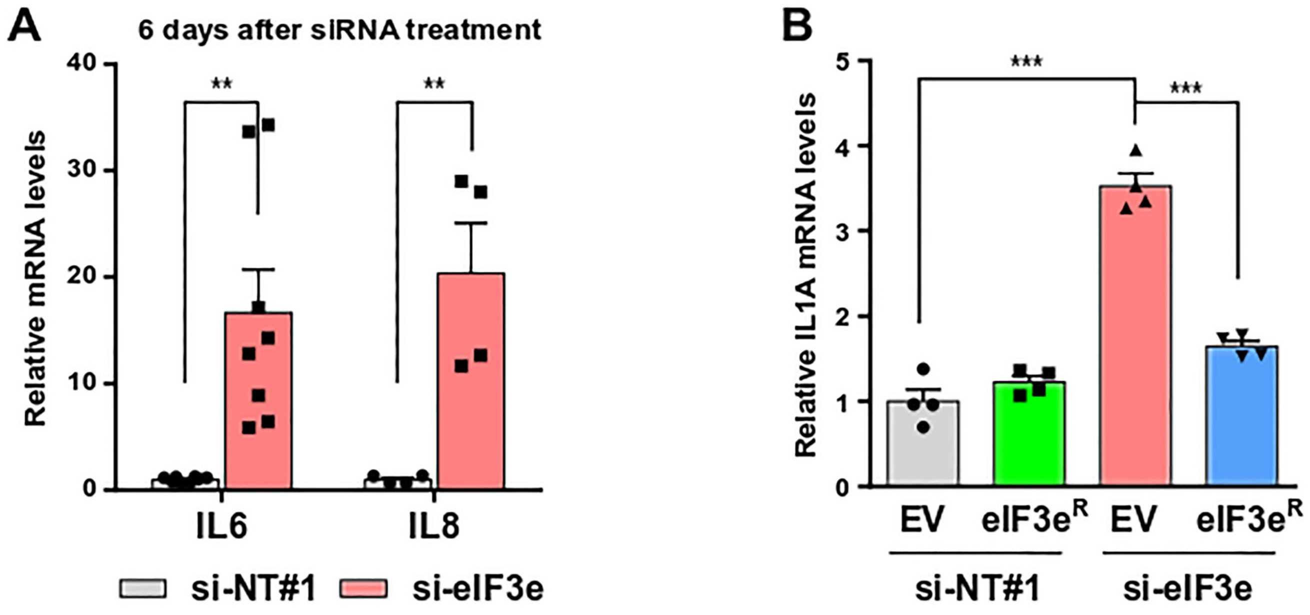

Supplementary Figure 6: eIF3e depletion promotes a SASP response. (**A**) Quantification of mRNAs encoding IL6 and IL8 from MDA-MB-231 cells transfected for 6 days with control or eIF3e siRNAs. Transcript levels were measured by RT-qPCR and a value of 1 was assigned to mRNA levels of control cells. $n = 8$ from 4 independent experiments for IL6 and $n = 4$ from 2 independent experiments for IL8. (**B**) eIF3e re-expression in eIF3e-depleted cells reverts the SASP. MDA-MB-231 cells were treated with siRNAs non-targeting or targeting eIF3e and, the day after, cells were transfected with an empty vector (EV) or a vector expressing an eIF3e cDNA (eIF3eR) resistant to degradation by the siRNA eIF3e. Cells were collected 3 days later for RNA extraction. IL1A mRNA levels were measured by RT-qPCR and a value of 1 was assigned to mRNA level of control cells. $n = 4$ from 2 independent experiments. Error bars represent means ± SEM. Statistical significances were calculated using unpaired t test, $^{***}P < 0.001$, $^{**}P < 0.01$.

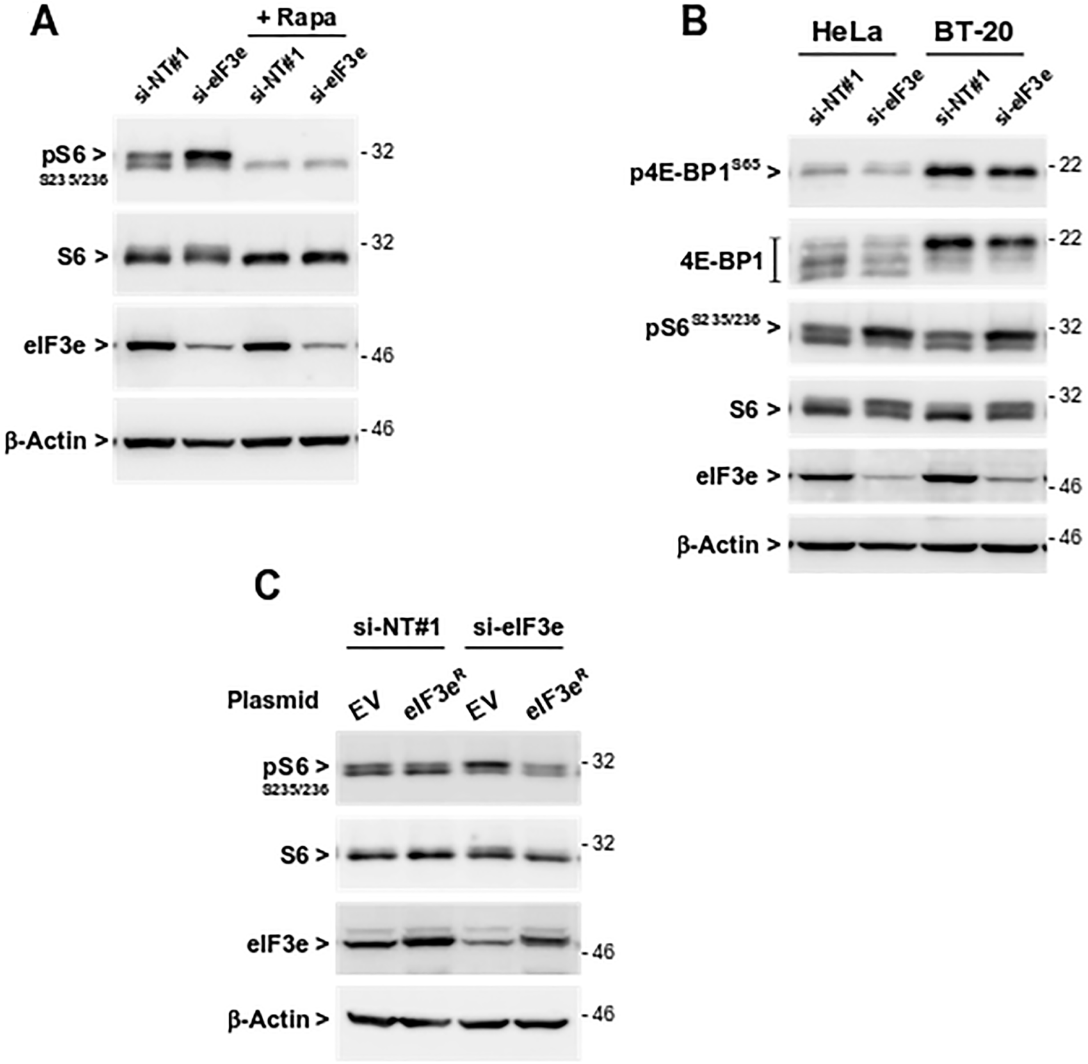

Supplementary Figure 7: eIF3e deficiency results in aberrant activation of the mTORC1-S6K1 signaling axis. (**A**) BT-20 cells were transfected with control or eIF3e siRNAs for 3 days, the last 2 days in the presence or absence of 50 nM of rapamycin. Phosphorylation of ribosomal protein S6 was assessed by immunoblotting with the indicated antibodies. RNA interference efficacy and protein loading were controlled by detection of eIF3e and β-actin, respectively. (**B**) HeLa and BT-20 cells were transfected with control or eIF3e siRNAs for 3 days. To monitor mTORC1 activity through its effectors 4E-BP1 and S6K1, immunoblots were carried out using the indicated antibodies. RNA interference efficacy and protein loading were checked as in (A). (**C**) eIF3e re-expression in eIF3e-depleted cells reverses the aberrant activation of S6 phosphorylation. MDA-MB-231 cells were treated with control or eIF3e siRNAs and, after 24 h, cells were transfected with the parental vector (EV) or the vector expressing an eIF3e cDNA ($eIF3e^R$) resistant to degradation by the eIF3e siRNA, for 3 more days. Immunoblots were performed with the indicated antibodies. Efficiency of eIF3e depletion and re-expression and equal protein loading were controlled by detection of eIF3e and β-actin, respectively.
